# Comparative study of excretory–secretory proteins released by *Schistosoma mansoni*-resistant, susceptible and naïve *Biomphalaria glabrata*

**DOI:** 10.1186/s13071-019-3708-0

**Published:** 2019-09-14

**Authors:** Conor E. Fogarty, Min Zhao, Donald P. McManus, Mary G. Duke, Scott F. Cummins, Tianfang Wang

**Affiliations:** 1 Genecology Research Centre, University of the Sunshine Coast, Maroochydore DC, QLD 4558 Australia; 2 QIMR Berghofer Medical Research Institute, Brisbane, QLD 4006 Australia

**Keywords:** *Schistosoma mansoni*, *Biomphalaria glabrata*, Miracidia, Excretory–secretory proteins, Proteomics, PPI

## Abstract

**Background:**

Schistosomiasis is a harmful neglected tropical disease caused by infection with *Schistosoma* spp., such as *Schistosoma mansoni*. *Schistosoma* must transition within a molluscan host to survive. Chemical analyses of schistosome-molluscan interactions indicate that host identification involves chemosensation, including naïve host preference. Proteomic technique advances enable sophisticated comparative analyses between infected and naïve snail host proteins. This study aimed to compare resistant, susceptible and naïve *Biomphalaria glabrata* snail-conditioned water (SCW) to identify potential attractants and deterrents.

**Methods:**

Behavioural bioassays were performed on *S. mansoni* miracidia to compare the effects of susceptible, F1 resistant and naïve *B. glabrata* SCW. The F1 resistant and susceptible *B. glabrata* SCW excretory–secretory proteins (ESPs) were fractionated using SDS-PAGE, identified with LC-MS/MS and compared to naïve snail ESPs. Protein-protein interaction (PPI) analyses based on published studies (including experiments, co-expression, text-mining and gene fusion) identified *S. mansoni* and *B. glabrata* protein interaction. Data are available *via* ProteomeXchange with identifier PXD015129.

**Results:**

A total of 291, 410 and 597 ESPs were detected in the susceptible, F1 resistant and naïve SCW, respectively. Less overlap in ESPs was identified between susceptible and naïve snails than F1 resistant and naïve snails. F1 resistant *B. glabrata* ESPs were predominately associated with anti-pathogen activity and detoxification, such as leukocyte elastase and peroxiredoxin. Susceptible *B. glabrata* several proteins correlated with immunity and anti-inflammation, such as glutathione S-transferase and zinc metalloproteinase, and *S. mansoni* sporocyst presence. PPI analyses found that uncharacterised *S. mansoni* protein Smp_142140.1 potentially interacts with numerous *B. glabrata* proteins.

**Conclusions:**

This study identified ESPs released by F1 resistant, susceptible and naïve *B. glabrata* to explain *S. mansoni* miracidia interplay. Susceptible *B. glabrata* ESPs shed light on potential *S. mansoni* miracidia deterrents. Further targeted research on specific ESPs identified in this study could help inhibit *B. glabrata* and *S. mansoni* interactions and stop human schistosomiasis.

## Background

Human schistosomiasis is caused by infection from digenetic trematodes of the genus *Schistosoma* and is one of the greatest threats to public health in the world [1, 2]. The disease is currently endemic in 76 different countries and over 800 million people are at risk of infection [3, 4]. Estimates claim that over 200,000 people die every year from the immunosuppressive and carcinogenic effects of the infection [5–7]. It decreases resistance to other harmful diseases including hepatitis B, HIV and malaria [8–10]. It also increases rates of seizures, infertility and anaemia [11, 12].

The chemotherapeutic drug praziquantel is the most effective current method of dealing with human schistosomiasis [13]. While it has a low cost of production and few side effects less than 30% of those in need of preventative chemotherapy had access to it in 2015 [14, 15]. Additionally, its decreased efficacy against immature schistosomes and reinfections necessitates the innovation of alternative methods for mitigating the spread of schistosomiasis [16, 17]. To meet the World Health Organization’s goal of eradicating the disease by 2025 the disruption of the parasites’ lifecycles in their infective stages is an approach currently being investigated [18].

As a member of the class Trematoda, *Schistosoma* must infect an intermediate molluscan host [19]. Molluscan hosts are infected by *Schistosoma* miracidia, non-feeding infective stages that hatch from eggs released from mammalian host defecation within minutes of entering fresh water [7, 20, 21]. Following penetration, the miracidia transform into primary (or mother) sporocysts which give rise asexually to a second generation of secondary (or daughter) sporocysts [22]. Secondary sporocysts may produce thousands of cercariae, resulting in infection from one miracidium potentially resulting in the release of more than 100,000 cercariae [23, 24]. *Schistosoma* cercariae are (a non-feeding infective stage) penetrate and reproduce within a host of the class Mammalia [20]. Among the species of *Schistosoma* which may infect humans, the most harmful are *Schistosoma mansoni*, *Schistosoma japonicum* and *Schistosoma haematobium*, which together comprise over 95% of human infections [25].

Miracidia can only survive in the environment for an average of 12 hours [21]. Analyses of the interactions between *S. mansoni* and one of its molluscan hosts, *Biomphalaria glabrata*, indicate that the miracidia locate the host through chemosensory signals [7]. The interactions between miracidia and their hosts’ immune recognition receptors render the host susceptible to infection [19]. Haemocytes kill the schistosomes through hydrogen peroxide exposure upon identification [26]. Successful infections from schistosomes lead to chemical castration and decreased average lifespans in molluscan hosts [16, 27].

Various haemocyte-related enzymes and receptors are upregulated within 5 hours after infection in more resistant strains (such as BS-90) while responses take several hours or days longer in susceptible strains of *B. glabrata* (such as NMRI) [28, 29]. At this stage there is insufficient information to determine the precise relationship between haemocyte behaviour and snail excretory–secretory proteins (ESPs). Furthermore, it is unclear how specific ESPs are to certain species or strains. However, the availability of genomes for the *S. mansoni* and *B. glabrata* allow for in-depth genomic and proteomic studies [16, 30]. Recently, studies have been conducted to identify the ESPs released by naïve *B. glabrata* [16, 31]. There remain some gaps regarding our understanding of the differences and significance of ESPs released by susceptible and resistant *B. glabrata*.

In this study we performed behavioural bioassays in conjunction with video analyses using SCW derived from naïve, susceptible and F1 resistant *B. glabrata* (NMRI strain). ESPs identified by LC-MS/MS analysis were further assessed for their potential roles as attractants and defensive proteins in parasite infection. A protein-protein interaction (PPI) analysis was used to determine potential interactions between identified *S. mansoni* proteins and its entire proteome, or *B. glabrata* proteome, respectively. The findings of this study help elucidate the ESPs related to resistance mechanisms of *B. glabrata*. This information may facilitate the development of synthetic attractants or deterrents of miracidia, mitigating the spread of schistosomiasis.

## Methods

### *Biomphalaria glabrata* maintenance conditions

*Biomphalaria glabrata* snails of the NMRI strain (which reliably release cercariae in up to 95% of infection cases) [32], were maintained in an aerated tank of calcium carbonate conditioned-water (pH-neutral) at 27 °C in a 12 h alternating cycle of light and darkness. Their diet consisted of algae tablets and lettuce. Naïve *B. glabrata* were defined as those with no prior exposure to *S. mansoni* miracidia. The resistant snails were defined as the F1 progeny of *B. glabrata* that were exposed to *S. mansoni* miracidia (stock). These offspring were expected to maintain the resistance of their parent and therefore have a relatively high probability of also being resistant [33]. Susceptible snails were those previously exposed to *S. mansoni* miracidia and rendered infertile, a key indicator of reproductive dysfunction due to infection.

### Snail conditioned water collection and semi-purification of biomolecules

**Fig. 1:**
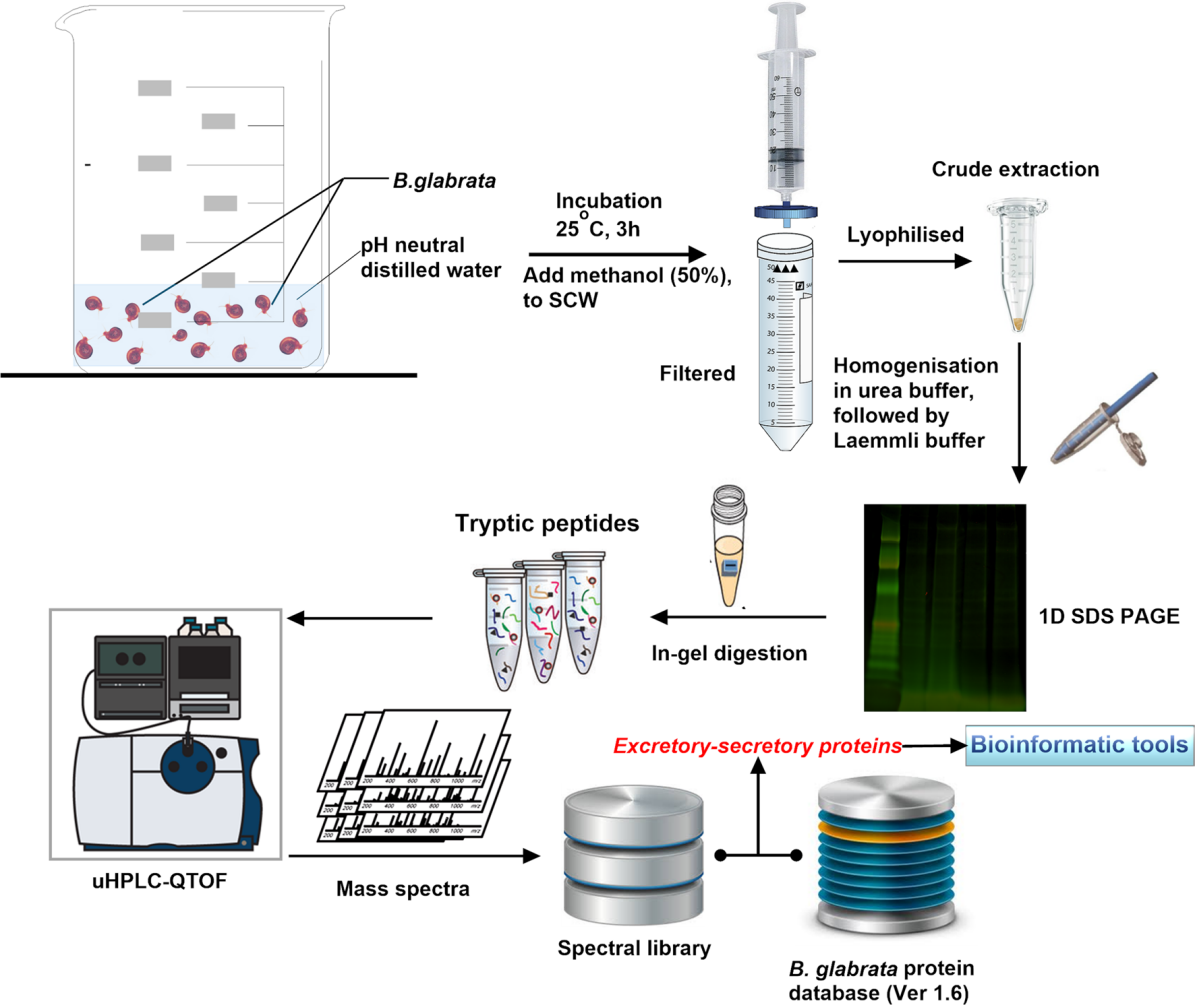
Overall workflow for *B. glabrata* SCW isolation, protein extraction and identification. SCW proteins were extensively fractionated by 1D SDS-PAGE followed by identification with high-accuracy uHPLC QTOF MS/MS. Biological triplicates were performed for each experimental condition

The overall experimental procedure to map and annotate ESPs released by naïve, susceptible (14 days post-infection) and F1 resistant *B. glabrata* is outlined in Fig. 1. At QIMR Berghofer Medical Research Institute, *B. glabrata* snails (50 each) were washed four times with freshly prepared carbonate conditioned Milli Q water to remove any contaminants from the tank and separated into two 200 ml beakers, each containing 25 snails (Fig. 1). Snails were incubated in 20 ml of pH-neutral spring water at room temperature for 2 h. Snails were removed and returned to the aquarium, 20 ml of methanol was added to the water samples and mixed thoroughly. The mixture was filtered through a 0.45 µm Durapore PVDF filter (Bedford, MA, USA) to remove contaminants. Filtered samples were immediately frozen on dry ice until lyophilisation using a Savant SpeedVac Concentrator (Thermo Fisher Scientific, MA, USA).

### *Schistosoma mansoni* miracidia isolation and behavioural bioassay

*Schistosoma mansoni-*infected Swiss mice were euthanised with CO_2_ gas and their livers were perfused with chilled phosphate-buffered saline (PBS) to collect the eggs of *S. mansoni*. Two infected mouse livers were sliced with scalpel blades and blended to a smooth consistency in 50 ml PBS. The mixture was centrifuged (2000×*g* at 4 °C for 10 s), the supernatant was removed, and pellet re-suspended in 50 ml chilled PBS. This step was repeated three times until the supernatant was transparent. The mixture was incubated in a measuring cylinder surrounded by black tape in pH-neutral water under a light for 2 h at room temperature. The top layer of the water was collected, and the average miracidia were counted under a microscope. The miracidia were concentrated through centrifuging the water at 5000×*g* for 15 min at 22 °C and the supernatant was removed. The method of behavioural bioassay has been described in detail elsewhere [31]. Briefly, miracidia water aliquots in 200 µl volumes were placed on a petri dish and monitored using an Olympus-CKX41 microscope (Olympus) equipped with an Olympus DPI Digital Microscope Camera DP22 (25 frames per second at 2.8-megapixel image quality). Miracidia behaviour was recorded and monitored for one minute, followed by one minute after the addition of 2 µl of SCW. This process was conducted nine times using naïve, susceptible (from *B. glabrata* exposed to miracidia 2 weeks prior) and F1 resistant *B. glabrata* SCW and one negative control (pH-neutral water used for incubating miracidia).

The susceptible, F1 resistant and control sample videos were analysed statistically using the method described previously [31]. Videos were split into pre-SCW and post-SCW segments and imported into ImageJ (fiji-win64). The miracidia were identified when they were within the field of view (FOV) and their velocity was calculated in pixel s^−1^ using the rolling mean subtraction method [34]. Employing the plugin for FIJI software [35], known as TrackMate [36], miracidia location was tracked in each frame along an x–y axis and the trajectories were interpolated. The MTrackJ plugin [37] was used to determine the average velocity, tortuosity (the track length to maximum displacement ratio), duration of presence and sum of tracks per min of miracidia presence for the pre-SCW and post-SCW segments. Due to constant overlapping of miracidia pathways in naïve SCW videos, heatmaps (showing the distribution density of miracidia) were constructed to compare the effects of susceptible, resistant and naïve SCW. The protocol for heatmap generation has been described elsewhere [34].

A two-way ANOVA test was used to calculate *P*-values and evaluate the significance of behavioural modifications in response to pH-neutral water, susceptible and F1 resistant SCW. The behavioural changes in swimming speed (velocity), tortuosity, number of miracidia entering the FOV and the time of duration of miracidia staying in the FOV within the defined duration were compared. A change was considered significant if the *P*-value < 0.05.

### SDS-PAGE, Coomassie staining and in-gel trypsin digestion

The protein concentrations of the lyophilised samples were measured by Nanodrop 2000c (Thermo Fisher Scientific, MA, USA) before being resuspended in 100 µl of 6 M urea and mixed with 100 µl of sample buffer [95% of 2× Laemmli buffer (BioRad Laboratories, Hercules, CA, USA) and 5% of β-mercaptoethanol]. The mixture was heated at 95 °C for 5 min and loaded onto a preconditioned 4–15% Mini-PROTEAN®TGX™ Precast Protein Gels (Bio-RAD). The gel was run in a Mini-PROTEAN® Dodeca Cell for 60 min under 200 V. The gel was stained using Coomassie brilliant blue G-250 for 1 h, rinsed in water for 30 min and placed in the fridge at 4 °C overnight. The image of the gel was scanned with the wavelengths of 700 nm for 40 min using an Odyssey CLx (Li-Cor) and visualised with Image Studio 4.0 (Li-Cor). The naïve *B. glabrata* gel was collected in an earlier study [16].

The entire gel lanes were excised into pieces using a scalpel blade and subjected to in-gel trypsin digestion as described elsewhere [38]. Briefly, the gel pieces were transferred to Eppendorf tubes and repeatedly washed in 500 µl of 50 mM NH_4_HCO_3_, incubated for 5 min and removed. A 500 µl volume of 50 mM NH_4_HCO_3_ in 30% acetonitrile was added to remove Coomassie stain. Pieces were incubated in a sonicating water bath for 15 min and centrifuged using pulse centrifugation before the excess liquid was extracted. A 200 µl volume of acetonitrile was added to each tube to shrink the gel pieces, incubated for 15 min and spun down using pulse centrifugation before the liquid was removed. Samples were vacuum-centrifuged for 10 min. Pieces were swelled with 50 µl of 10 mM dithiothreitol in 100 mM NH_4_HCO_3_ before being incubated at 56 °C for 1 h. Samples were spun down using pulse centrifugation after cooling to room temperature and the excess liquid was extracted. A volume of 200 µl of acetonitrile was added to each sample, incubated for 15 min and pulse-centrifuged before the liquid was removed. A 50 µl volume of 55 mM iodoacetamide in 100 mM NH_4_HCO_3_ was added and the pieces were incubated in the dark for 45 min. The solution was removed, 100 µl of 5 mM NH_4_HCO_3_ was added and incubated for 10 min before being removed. The pieces were shrunk using 200 µl of acetonitrile and incubated from 15 min before pulse centrifugation. The liquid was removed, and the gel pieces were vacuum centrifuged for 10 min. A 10 µl aliquot of 10 ng/µl trypsin in 5 mM NH_4_HCO_3_ was added to each tube and incubated overnight (~16 h) at 37 °C.

The tryptic peptides were extracted from gel pieces by sonication in a water bath for 15 min after adding 20 µl of 50% acetonitrile containing 1% formic acid. The samples were spun down by pulse centrifugation and the excess liquid was transferred into the final sample tube. The gel pieces were shrunk by 50 µl of 100% acetonitrile and the liquid was collected into the corresponding tubes after 15 min sonicating in a water bath. The volume of the liquid in each tube was reduced to about 1 µl with SpeedVac and transferred into the final sample tube. This was reconstituted in 5 µl of 30% acetonitrile, 0.1% formic acid and stored at − 20 °C for LC-MS/MS analysis (see Fig. 1).

### uHPLC tandem QTOF MS/MS analyses

Tryptic peptides were resuspended in 25 μl of 1% formic acid in MilliQ water and analysed by LC-MS/MS attached to an ExionLC liquid chromatography system (AB SCIEX, Concord, Canada) and a QTOF X500R mass spectrometer (AB SCIEX, Concord, Canada) equipped with an electrospray ion source. A 20 µl sample of each of the *B. glabrata* fractions was injected into a 100 mm × 1.7 μm Aeris PEPTIDE XB-C18 100 uHPLC column (Phenomenex, Sydney, Australia) equipped with a SecurityGuard column for mass spectrometry analysis. Linear gradients of 5–35% solvent B over a 10-min period at a flow rate of 400 µl/min, followed by a gradient from 35% to 80% solvent B over 2 min and 80% to 95% solvent B in 1 min were used for peptide elution. Solvent B remained at 95% for a 1 min period for washing the column after which it was decreased to 5% for equilibration prior to the injection of the subsequent sample. Solvent A consisted of 0.1% formic acid in MilliQ water while solvent B contained 0.1% formic acid in 100% acetonitrile. The ion spray voltage was set to 5500 V, the declustering potential was set to 100 V, the curtain gas flow was set at 30, ion source gas 1 was set at 40, the ion source gas 2 was set at 50 and spray temperature was set at 450 °C. The mass spectrometer acquired the mass spectral data in an Information Dependant Acquisition, IDA mode. Full scan TOFMS data was acquired over the mass range of 350–1400 and for product ion ms/ms of 50–1800. Ions observed in the TOF-MS scan exceeding a threshold of 100 cps and a charge state of + 2 to + 5 were set to trigger the acquisition of product ion. The data were acquired and processed using SCIEX OS software (AB SCIEX, Concord, Canada).

### Protein identification

LC-MS/MS data was imported to PEAKS studio (Bioinformatics Solutions Inc., Waterloo, ON, Canada, version 7.0) with the assistance of MSConvert module of ProteoWizard (3.0.1) [39]. The ESPs of naïve *B. glabrata* have been analysed using a similar protocol with a previous version the genome (Ver 1.2) [16]. For the present study, the proteomic data were reanalysed with the most up-to-date database (BglaB1.6) (see Additional file 1: Database S1) to provide a better comparison between naïve *B. glabrata* ESPs and those released by the susceptible and resistant snails (https://www.vectorbase.org/organisms/biomphalaria-glabrata) [16]. Meanwhile, MS/MS spectra of proteins extracted from susceptible *B. glabrata* conditioned water were analysed with reference to the *S. mansoni* protein database (https://parasite.wormbase.org/Schistosoma_mansoni_prjea36577/Info/Index). *De novo* sequencing of peptides, database search and characterising specific PTMs were used to analyse the raw data; false discovery rate (FDR) was set to ≤ 1%, and [− 10*log(p)] was calculated accordingly where p was the probability that an observed match was a random event. The PEAKS used the following parameters: (i) precursor ion mass tolerance, 0.1 Da; (ii) fragment ion mass tolerance, 0.1 Da (the error tolerance); (iii) tryptic enzyme specificity with two missed cleavages allowed; (iv) monoisotopic precursor mass and fragment ion mass; (v) a fixed modification of cysteine carbamidomethylation; and (vi) variable modifications including lysine acetylation, deamidation on asparagine and glutamine, oxidation of methionine and conversion of glutamic acid and glutamine to pyroglutamate. The mass spectrometry proteomics data have been deposited to the ProteomeXchange Consortium via the PRIDE [40] partner repository with the dataset identifier PXD015129.

### Prediction of secreted proteins, gene ontology and KEGG pathway analysis

Identified proteins were subjected to BLASTp using non-redundant protein sequences of NCBI. Protein N-terminal signal sequences were predicted using SignlaP 4.1 [41] and Predisi [42], with the transmembrane domains predicted by TMHMM [43]. For SignalP predictions, positive identifications were made when both neural network and hidden Markov model algorithms gave coincident estimations; D-cut-off values were set to 0.34 (to increase sensitivity) for both SignalP-noTM and TM networks. Herein, a protein was designated as secreted only when it met the criteria of both SignalP and Predisi and did not have a transmembrane domain predicted by TMHMM.

BLAST results were combined and imported to BLAST2GO [44] (version 5.1), to perform gene ontology (GO) and KEGG pathway analysis. Fisher’s extract test was carried out to evaluate the enrichment of GO terms in ESPs of susceptible and resistant snails with reference to entire proteome of *B. glabrata* [45]. Susceptible *B. glabrata* SCW was also referenced with respect to the *S. mansoni* proteome. The significant GO terms with *P *< 0.01 were considered as over-represented, and FDRs were calculated from p-values using the Benjamini–Hochberg procedure [46].

### Protein-protein interaction (PPI) network

We investigated the PPI maps following a similar procedure reported elsewhere [47]. Both domain-domain interaction and gene ontology annotations were used. Briefly, HMMER [48] was first used to annotate all the known protein domains based on the Pfam database (Release 32.0) [49], then the high confidence domain-domain interactions from the DOMINE database [50] was exported based on annotations. Proteins with at least three domain-domain interaction supports were included in the final network. The PPI between annotated *S. mansoni* proteins identified in susceptible snail SCW and its entire proteome was further validated with STRING [51]. STRING integrates protein-protein interactions from multiple resources, including direct (physical) as well as indirect (functional) associations. All resources were selected to generate the network and ‘confidence’ was used as the meaning of network edges. The first shell was set to show no more than 20 interactors, while the second shell was not considered in this study. Proteins without any interaction with other proteins were excluded from the network of this study. Topological analyses were performed to explore the potential functions in our constructed network using the Network Analyzer plugin in Cytoscape 3.7.1 [52]. The final network visualization was performed using Cytoscape [52].

## Results

### *Schistosoma mansoni* miracidia behavioural assays

**Fig. 2:**
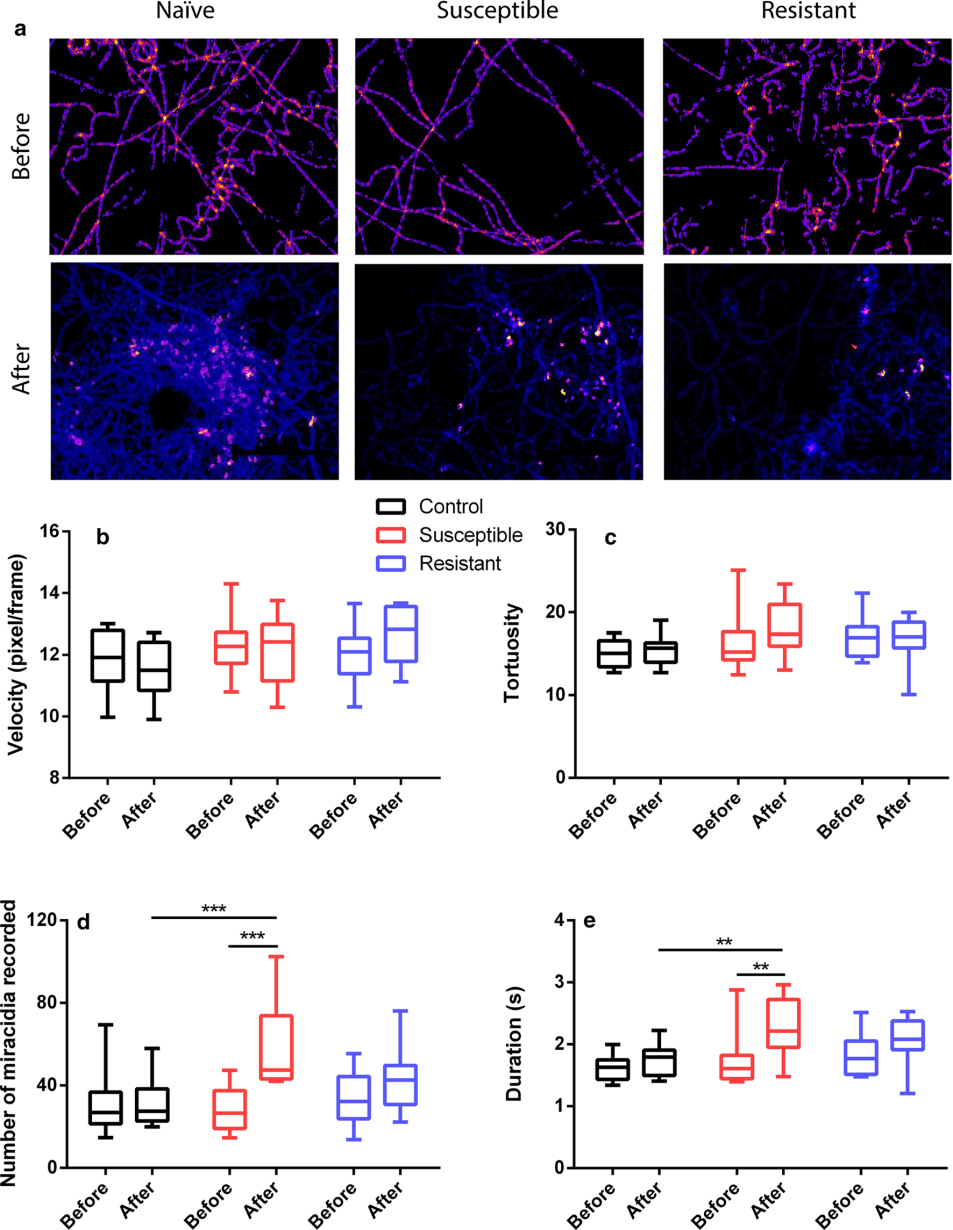
Behavioural modifications of *S. mansoni* miracidia before and after exposure to pH-neutral water, susceptible and resistant SCW. The heatmaps (**a**), linear velocity (**b**), tortuosity (**c**), number of miracidia (**d**) recorded in the FOV within 1 min pre- and post- the addition and duration of miracidia staying in the FOV within 1 min pre- and post- the addition (**e**). A two-way ANOVA test was used to calculate *P*-values: **P < 0.01, ***P < 0.001

We have previously shown that SCW of naïve *B. glabrata* stimulates significant behavioural changes in *S. mansoni* miracidia, including the elevation of swimming speed (velocity), tortuosity, number of miracidia entering the FOV and the time of duration of miracidia staying in the FOV within a defined time period [31]. In this study, we further quantified the changes in *S. mansoni* miracidia behaviour in response to pH-neutral water, susceptible and resistant *B. glabrata* SCW using behavioural bioassays. Figure 2 provides comparative data for the behavioural modifications monitored in the bioassay, with the statistical analysis results shown in Additional file 2: Table S1. Figure 2a displays there are more abundant red and yellow regions in pre-addition heatmaps, indicating relatively slower moving miracidia. The post-addition heatmap of naïve SCW depicts a significantly fewer linear motions and higher proportion of soft blue lines which suggest more tracks in the FOV and quicker circular movements. The post-addition heatmaps of susceptible and F1 resistant SCW only show quicker circular movements, but the changes in the amount of blue lines seems insignificant. The velocity of movement (swimming) of miracidia in three treatments was assessed (Fig. 2b), where no significant difference could be determined between the treatments (i.e. pH-neutral water versus susceptible versus resistant), or with one treatment (i.e. pre- *versus* post- addition within 1 min time frame) (Additional file 2: Table S1). In terms of tortuosity, the behavioural change was also determined to be insignificant within one treatment or between treatments, although the mean value post-treatment of susceptible SCW increased (Fig. 2c). The number of miracidia entering the FOV significantly increased within 1 min post-addition of susceptible SCW, but not after addition of pH-neutral water or resistant SCW, which also showed a higher average number compared to that observed pre-addition (Fig. 2d). The duration of miracidia staying in the FOV was only found to be significantly elevated after the addition of susceptible SCW, while the resistant SCW presented an insignificantly increased mean duration (Fig. 2e).

### *Biomphalaria glabrata* ESP proteomic analysis

**Fig. 3:**
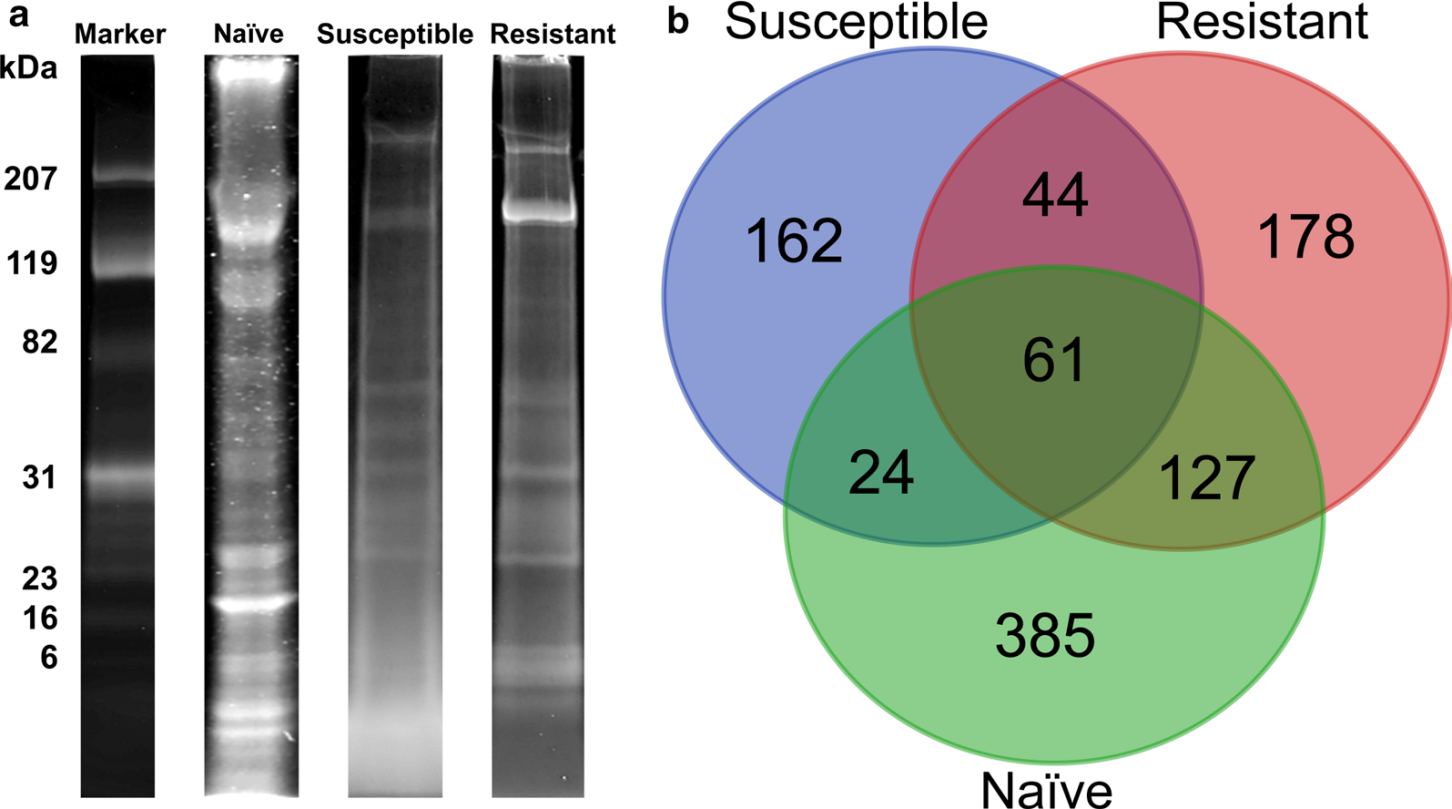
Comparison of 1D SDS-PAGE (with Coomassie stain) images and the number of ESPs identified by LC-MS/MS. **a** Representative gel images of three experimental conditions and molecular weight marker (kDa). **b** Venn diagram showing number of ESPs identified in SCW from naïve, infected and resistant *B. glabrata*

ESPs from susceptible, resistant and naïve snails were fractionated using 1D SDS-PAGE and visualised by Coomassie Blue staining. Figure 3a provides a representative gel lane of the different SCW conditions showing visibly distinct differences that suggest the contents and abundance of the proteins released vary considerably. Naïve SCW presented a relatively higher abundance of ESPs at high molecular weight (>~100 kDa). Conversely, ESPs of susceptible snails are mainly distributed in middle-to-low molecular weight regions (~30–6 kDa) and the distribution of intense bands was lower compared to those of naïve and resistant SCW. Of the two intense bands observed in resistant SCW ESPs (~ 207 kDa), the lower band was more prominent. This differed from the naïve and susceptible SCW ESPs. ESPs were also more common in the middle molecular weight region (~60–23 kDa) in resistant compared to susceptible SCW ESPs (Fig. 3a).

All samples were subjected to high-accuracy mass spectrometry and the raw data were rigorously analysed using available informatics tools. Details of all 981 ESPs identified, including ID number, best BLAST match and MS peptide matches, are provided in Additional files 3, 4, 5: Tables S2-S4. Naïve SCW provided the highest number of ESPs (597; Fig. 3b), which represented almost 61% of all identified proteins. SCW obtained from resistant and susceptible snails revealed 410 and 291 ESPs, respectively (Fig. 3b). A comparison of ESPs identified in resistant, susceptible and naïve *B. glabrata* revealed that 61 (approximately 6%) of the 981 proteins identified are shared. There was most overlap between the proteins expressed by the resistant and naïve snails (188) and less than half that between the susceptible and naïve snails (85). The ESPs uniquely identified within the three conditions were 162, 178 and 385 from susceptible, F1 resistant and naïve SCW, respectively.

### *Biomphalaria glabrata* ESP annotation and gene ontology analysis

The identified ESPs were annotated using BLAST against the reference database of NCBI. Several ESPs identified in naïve SCW with high confidence MS/MS spectra were enzymes, such as superoxide dismutase (SOD), leukocyte elastase and dipeptidase (Additional file 3: Table S2). Several others were associated with the dermis, including microtubule-related proteins and lamin derivatives. Multiple ESPs supported by high confidence MS/MS spectra were annotated within resistant *B. glabrata* SCW. This included carboxypeptidase, glutathione peroxidase, SOD and adenosine deaminase (Table 1). The full list of proteins is provided in Additional file 4: Table S3.

**Table 1:** High confidence non-redundant ESPs ([− 10lgP] > 30) with putative anti-pathogen function identified in resistant *B. glabrata* SCW. Details of coverage, peptide match number and BLAST confidence (e-value) are shown

Description	− 10lgP	Coverage (%)^a^	No. of peptides	No. unique^b^	Avg. mass	BLASTp e-value	Accession No.
Leukocyte elastase inhibitor-like	73.93	15	2	1	15593	1.01E−98	BGLB007529-PB
Haemoglobin type 1	69.56	12	4	1	20380	4.65E−130	BGLB026333-PA
Superoxide dismutase [Cu-Zn]	59.07	14	2	2	16447	4.39E−104	BGLB000035-PA
Adenosine deaminase CECR1	58.36	8	2	2	25076	3.34E−159	BGLB033953-PA
Glutathione peroxidase-like	50.23	12	2	2	22859	1.85E−151	BGLB008980-PC
Heat-shock 70 kDa protein cognate 4	45.56	5	4	4	71177	0	BGLB007783-PB
Enolase-phosphatase E1	42.13	13	2	2	19313	4.05E−119	BGLB029757-PA
Peroxiredoxin 1	39.55	4	1	1	27567	0	BGLB000120-PA
Rho GDP-dissociation inhibitor 1	32.87	4	1	1	23176	2.75E−143	BGLB011409-PB
Carboxypeptidase B	32.85	12	1	1	12298	2.51E−73	BGLB037596-PA
Putative tyrosinase-like protein tyr-1	31.61	2	1	1	67700	0	BGLB030046-PA

^a^The whole protein sequence coverage from the peptides identified with LC-MS/MS

^b^The number of identified peptides unique to the protein

*Abbreviations*: No., number; avg, mean value

ESPs of [-10lg*P*] value greater than 30 identified in SCW of susceptible snails are detailed in Table 2. This included 23 non-redundant characterised ESPs, considerably fewer than those identified in naïve (132) or F1 resistant SCW (42) with similar cut-off *P-*value. Many high confidence ESPs were enzymes with extremely low e-values. Structural proteins, including collagen alpha-3(VI) chain isoform X1, microtubule-actin cross-linking isoform and tropomyosin, were present. Some uncertainty exists in the identification of *B. glabrata* glyceraldehyde-3-phosphate dehydrogenase (G3PDH), since the only supporting peptide detected also matches the peptide segment of *S. mansoni* G3PDH (Additional file 6: Figure S1). This is highlighted in Table 2 and was not included in the functional analysis due to this uncertainty. A complete list of proteins identified in the susceptible snails is provided in Additional file 5: Table S4.

**Table 2:** High confidence non-redundant ESPs (-10lgP > 30) identified in the susceptible *B. glabrata* SCW. Details of coverage, peptide match number and BLAST confidence (e-value) are shown

Description	− 10lgP	Coverage (%)^a^	No. of peptides	No. unique^b^	Avg. mass	BLASTp e-value	Accession No.
Zinc metalloproteinase/disintegrin-like	178.26	42	21	20	51345	0	BGLB040280-PA
Endothelin-converting enzyme 1-like isoform X2	111.23	11	6	6	56937	0	BGLB029484-PA
DUF4347 domain-containing protein	105.24	34	4	4	24685	9.89E−168	BGLB031575-PA
Solute carrier family 35-member E4	99.19	26	3	3	14320	8.17E−89	BGLB032352-PA
Proactivator polypeptide-like	91.36	8	6	5	68048	0	BGLB024213-PA
Probable serine carboxypeptidase CPVL	84.31	9	4	4	51401	0	BGLB030391-PA
Phospholipase B1, membrane-associated-like	79.92	8	3	3	44658	0	BGLB030448-PB
Chitinase-like protein PB1E7.04c	78	4	4	2	117976	0	BGLB017725-PA
Endothelin-converting enzyme 2-like	73.3	13	2	2	17072	5.67E−106	BGLB034604-PA
Cathepsin L1-like	39.16	7	2	2	26721	0	BGLB006210-PB
Collagen alpha-3(VI) chain-like isoform X1	35.7	3	1	1	34307	0	BGLB016458-PA
Probable glutathione S-transferase 7	35.27	8	2	2	23436	9.5E−150	BGLB004187-PC
Mucin-like protein	34.76	0	1	1	200228	0	BGLB016329-PA
Bypass of stop codon protein 1-like	34.45	7	1	1	16031	8.96E−99	BGLB034270-PA
ADAM family mig-17-like	32.27	2	1	1	51159	0	BGLB040281-PA
Glyceraldehyde-3-phosphate dehydrogenase^c^	27.37	3	1	1	25914	1.03E−176	BGLB010592-PB

^a^The whole protein sequence coverage from the peptides identified with LC-MS/MS

^b^The number of identified peptides unique to the protein

^c^Presence was confirmed from SCW using *S. mansoni* protein database (see Table 3 and Additional file 7: Figure S2), though its presence from the *B. glabrata* database is unconfirmed

*Abbreviations*: No., number; avg, mean value

**Fig. 4:**
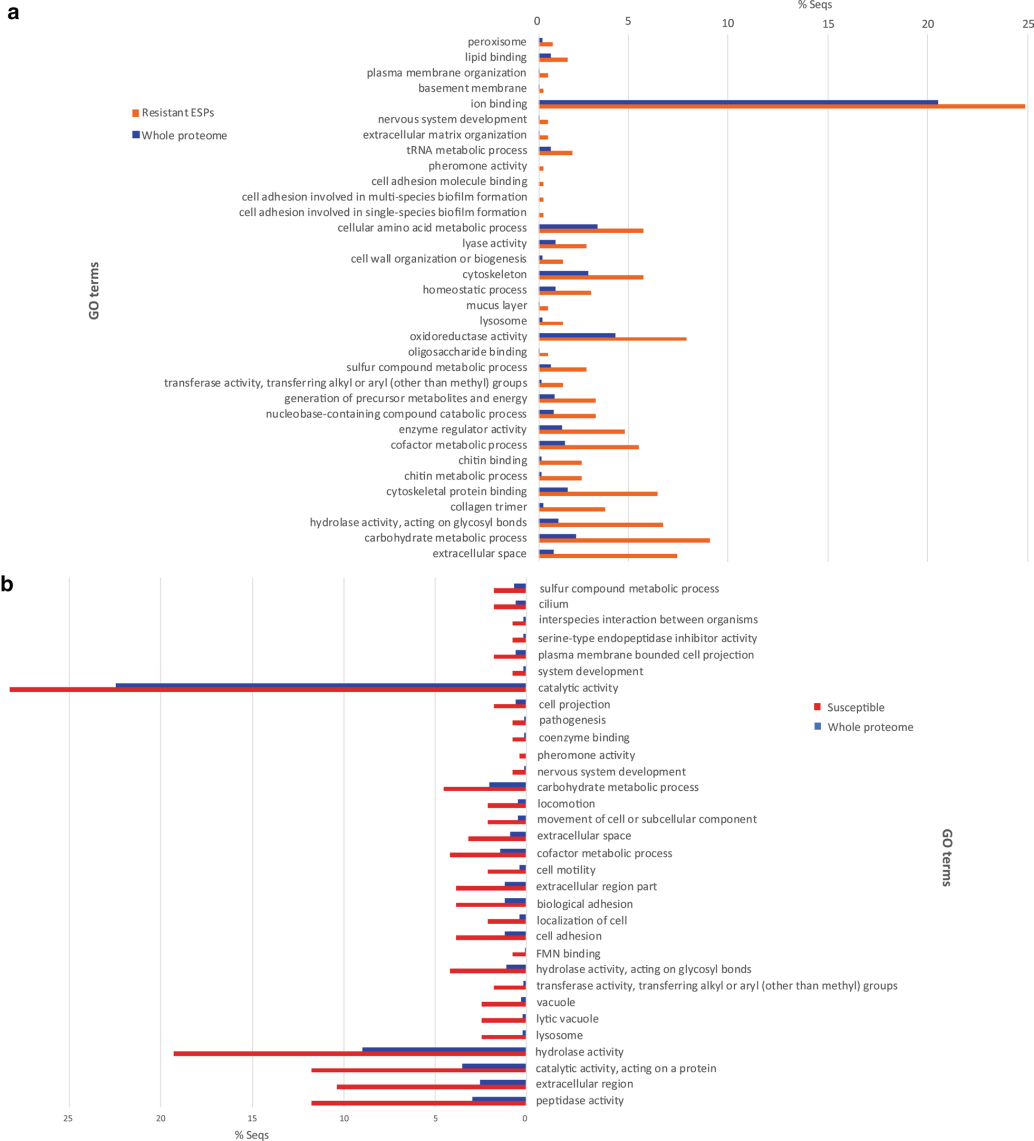
Gene ontology enrichment analysis of ESPs identified by LC-MS/MS in the SCW samples of susceptible and resistant *B. glabrata*. **a** The GO terms enriched in resistant *B. glabrata* ESPs. **b** The GO terms enriched in infected *B. glabrata* ESPs. The *B. glabrata* genome-derived proteome was used as the reference set in the analysis and *P*-value was set to below 0.05

Figure 4 provides comparative data for GO enrichment of resistant and susceptible SCW ESPs against the whole *B. glabrata* proteome. More than 20% of resistant ESPs potentially have the molecular function of ion-binding (Fig. 4a), while ‘catalytic activity’ corresponds to nearly 30% of susceptible ESPs (Fig. 4b). Other enriched GO terms in resistant ESPs include metabolic process of cellular amino acids, carbohydrates, cofactors, chitin, sulphur compounds and nucleobase-containing compounds, cytoskeletal and certain enzyme activities (Fig. 4a). For susceptible ESPs, hydrolase, peptidase, catalytic and extracellular activity were also enriched significantly (Fig. 4b). The differences in ESP GOs and their enrichment in comparison to whole proteome are an indicator that different types of processes were activated between resistant and susceptible snails.

### *Schistosoma mansoni* proteins identified from susceptible SCW of *B. glabrata*

The mass spectral data of susceptible *B. glabrata* SCW proteins were analysed using the *S. mansoni* protein database to identify *S. mansoni* proteins. Thirteen non-redundant *S. mansoni* proteins were supported by at least one unique peptide with high confidence (Table 3). The MS/MS spectra of supporting peptides of each protein are shown in Additional file 7: Figure S2 which showed at least five consecutive *b-* or *y-*ions, confirming the high confidence of these spectra for identification. These *S. mansoni* proteins include egg protein CP391B-like, G3PDH, putative nicotinate phosphoribosyltransferase and transcription factor TFIIF-alpha (Additional file 8: Table S5). Of the *S. mansoni* proteins two uncharacterised proteins (Smp_179420.1 and Smp_202190.1) had three and two respective unique peptides. A SignalP analysis predicted that Smp_179420.1 and Smp_093980.1 contain signal peptides (Additional file 8: Table S5).

**Table 3:** A list of non-redundant *S. mansoni* ESPs identified in susceptible SCW. Details of sequence coverage, average mass, number of peptides and unique peptides and accession names are shown

Description	− 10lgP	Coverage^a^ (%)	No. of peptides	No. unique^b^	Avg. mass	BLASTp e-value	Accession No.
Uncharacterised protein	66.61	8	3	3	31951	0	Smp_179420.1
Egg protein CP391B-like	54.01	6	1	1	25520	4.00E−157	Smp_193380.1
Glyceraldehyde-3-phosphate dehydrogenase	44.08	7	3	1	33808	0	Smp_056970.4
Putative zinc finger protein	37.91	1	3	1	286355	0	Smp_132000.1
Uncharacterised protein	28.51	19	2	2	7535	3.00E−41	Smp_202190.1
Uncharacterised protein	21.49	1	1	1	228220	0	Smp_198230.1
Uncharacterised protein	21.43	2	1	1	48135	0	Smp_137420.1
Putative nicotinate phosphoribosyltransferase	20.87	1	1	1	60943	0	Smp_035460.1
Tfiif-alpha	20.19	1	1	1	92315	0	Smp_088460.1
Uncharacterised protein	20.01	4	1	1	28284	0	Smp_093980.1
Uncharacterised protein	19.55	2	1	1	47835	0	Smp_130330.1
Proline–serine–threonine phosphatase interacting protein	19.54	1	1	1	141331	0	Smp_028140.1
Uncharacterised protein	18.4	1	1	1	83862	0	Smp_142140.1

^a^The whole protein sequence coverage from the peptides identified with LC-MS/MS

^b^The number of identified peptides unique to the protein

*Abbreviations*: No., number; avg, mean value

**Fig. 5:**
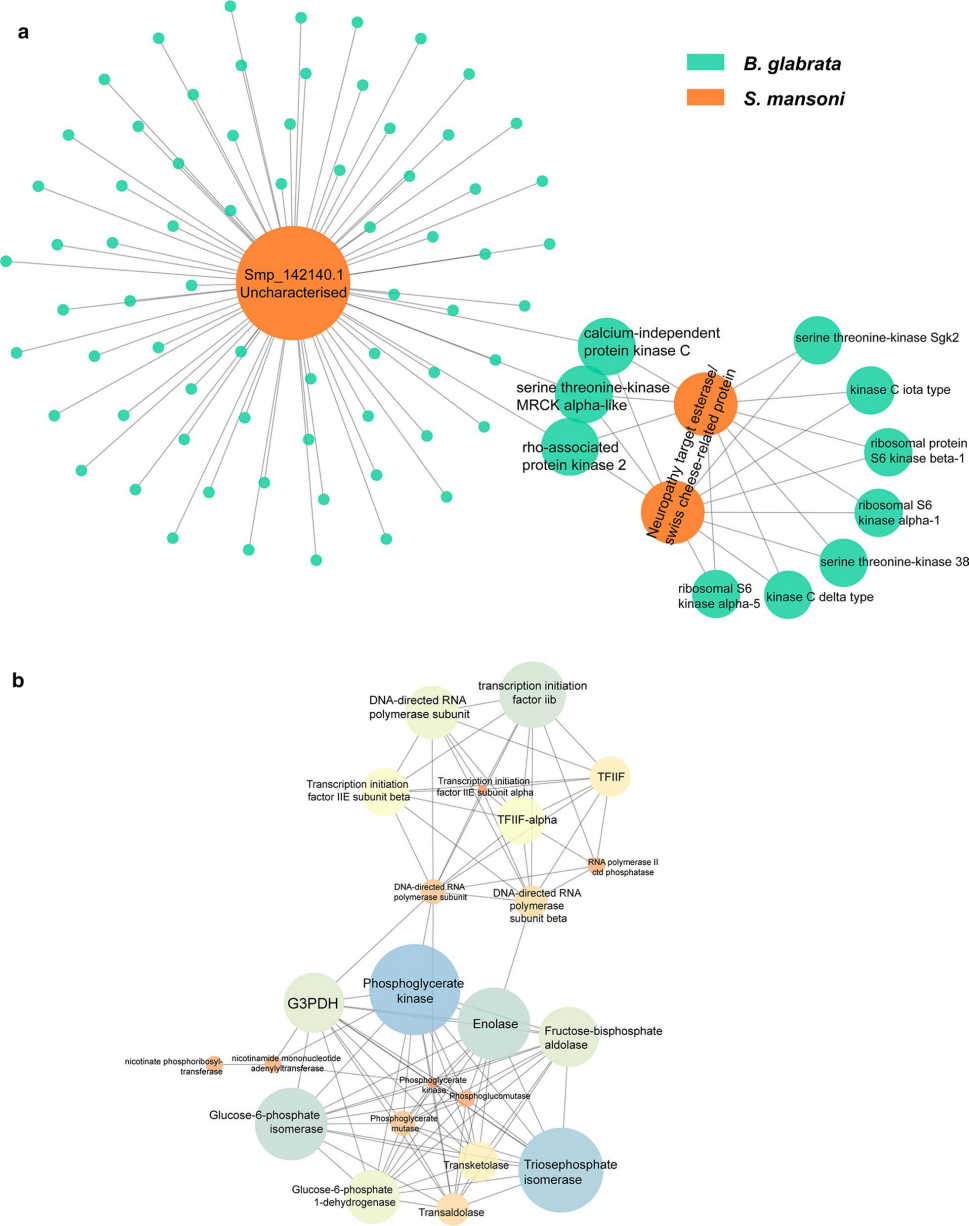
Identification of protein-protein interactions using susceptible ESPs. The PPIs of *S. mansoni* proteins identified in susceptible SCW with the whole *B. glabrata* proteome (Ver 1.6) (**a**) and with the whole *S. mansoni* proteome (**b**). In **b**, PPIs were filtered by STRING to only include those with experimental support. The size of the node represents the degree of interaction

Protein-protein interaction (PPI) networks were constructed between identified *S. mansoni* proteins (see Table 3) and the *B. glabrata* genome-derived proteome (Fig. 5a), with node proteins shown in Additional file 9: Table S6. Smp_142140.1 was identified to interact with numerous *B. glabrata* proteins, of which major nodes include the calcium-independent protein kinase C (PKC), rho-associated protein kinase 2 and serine threonine-kinase MRCK alpha-like proteins. These three proteins were noted to each interact with *S. mansoni* neuropathy target esterase/Swiss cheese-related protein This protein interacts with seven other *B. glabrata* proteins, including two PKCs, two serine threonine-kinase and three ribosomal proteins. There are 70 *B. glabrata* proteins that interact exclusively with Smp_142140.1, such as 1-phosphatidylinositol 4,5-bisphosphate phosphodiesterase, calpain, mitogen-activated kinase and spermine oxidase (Additional file 9: Table S6). The BLASTp annotation of Smp_142140.1 predicts the existence of an EF-hand domain (helix-loop-helix structure domain).

Figure 5b indicates that several identified *S. mansoni* proteins may interact with many other proteins of the *S. mansoni* proteome. G3PDH interacts with several enzymes playing important roles in metabolism of *S. mansoni*, including phosphoglycerate kinase, glucose-6-phosphate isomerase and phosphoglycerate mutase. Another highly connected node, TFIIF-alpha, interacts with various RNA polymerase subunits and transcription initiation factor proteins. Many of these interactions were supported by the co-expressions of their corresponding genes within *S. mansoni* and/or other model organisms suggested by STRING (Additional file 10: Figure S3). The enrichment analysis of this PPI identifies 107 edges among 33 nodes, which shows significantly more interactions (*P-*value* < *1.0e-16). Enriched biological processes includes a few metabolic processes, such as carbohydrate metabolic, pyruvate metabolic, glycolytic, nucleotide catabolic and glucose metabolic processes (false discovery rate, FDR* < *0.0001), associated KEGG pathways (FDR* < *0.0001) are carbon metabolism, glycolysis/gluconeogenesis, biosynthesis of amino acids, pentose phosphate pathway and basal transcription factors (Additional file 9: Table S6).

## Discussion

This study aimed to compare the ESPs of *Schistosoma*-resistant, susceptible and naïve *B. glabrata*, to shed light on the changes of ESPs that may lead to significantly different behavioural modifications of *S. mansoni* miracidia. This involved the collection, behavioural bioassay analysis, fractionation and comparison of SCW ESPs from these three groups. Additionally, PPIs were conducted to provide further information into the potential functions and significance of several of these proteins regarding infection.

In the presence of SCW, miracidia tend to increase their angular velocity while slightly decreasing linear velocity [31, 53]. We had previously shown that naïve *B. glabrata* SCW significantly reduced their velocity and elevated the tortuosity by approximately 20% and 70%, respectively [31]. As shown in Fig. 2b, c, none of the pH-neutral water, susceptible or F1 resistant *B. glabrata* SCW produced any significant change in miracidia linear velocity or tortuosity. The variation of velocity among post-SCW susceptible or F1 resistant samples is wider than those of pre-SCW, suggesting that SCW influences miracidia behaviour differently to naïve SCW [31]. The response of miracidia to pH-neutral water is consistent with expectations, as this water had not been exposed to any *B. glabrata*. The quantity of miracidia present following addition of susceptible SCW increased significantly when compared to pre-SCW or post-pH-neutral water addition, indicating a possible attraction effect (Fig. 2d). However, the increase in activity was remarkably weaker than that of naïve SCW, which produced an increasing magnitude of about 4-fold [31]. This suggests that susceptible *B. glabrata* at two-week post-infection might still release attractant(s) yet at a much lower concentration. A similar change was observed for the duration of miracidia staying in the FOV. In contrast, there was no significant change in miracidia quantity post-resistant SCW addition, possibly due to decreased attractant concentration compared to susceptible *B. glabrata*, or counteraction from potential repellents. This requires more experimental verification in future studies.

Our GO enrichment analysis of resistant *B. glabrata* ESPs revealed that some noticeable activities were related to oxidoreductase activities (see Fig. 5a). This includes SOD, which catabolises superoxide radicals to hydrogen peroxide that haemocytes commonly employ to kill schistosomes [54, 55]. Highly resistant strains, such as 13-16-R1 *B. glabrata*, tend to express higher concentrations of SOD than less resistant snails [56]. Some identified detoxifying agents for hydrogen peroxide include glutathione peroxidase and peroxiredoxin [57, 58]. Their functions involve the oxidation of hydrogen peroxide to water to prevent phospholipid peroxidation [59, 60]. Peroxiredoxin 1 and 2 have been identified to maintain molluscan health through catalysing the interaction between thioredoxin and hydrogen peroxide and are also expressed earlier in resistant strains than susceptible ones following infection [60, 61]. Hydrogen peroxide-detoxifying protein concentrations positively correlate to the snail’s resistance [60]. The detection of several different redox reactants and enzymes in the resistant snail ESPs is predominately consistent with the existing literature on intramolluscan defence mechanisms.

Other identified ESPs involved in parasite defence or immune protection include leukocyte elastase, tyrosinase, heat shock proteins and adenosine deaminase. The presence of leukocyte elastase is expected in resistant *B. glabrata*, as elastase has been identified to work in conjunction with hydrogen peroxide in terminating schistosome invaders [62]. Similarly, tyrosinase is suspected to be involved in anti-pathogen activity [63]. The upregulation of heat-shock protein is a common response in *B. glabrata* subjected to stress, such as molluscicide exposure and infection [64]. Abundance of adenosine deaminase has been noted in definitive hosts after taking praziquantel, suggesting anti-pathogen functions [65].

Carboxypeptidase’s presence suggests that resistant *B. glabrata* are healthier due to its functions as a digestive enzyme [66]. Identifying collagen may not be relevant because although it is present within proximity to haemocytes, it is not consistently affected by *S. mansoni* infection [67]. It may be an indicator of increased cell sloughing. Rho-GDP dissociation inhibitor has been associated with cytoskeletal formation [68]. Its direct immunological significance in *B. glabrata* is unknown. Haemoglobin has been suspected to play some role in pathogen resistance, though its function in *B. glabrata* immunology is still in need of further investigation [69].

Superoxide dismutase, peroxidase and collagen were also identified in naïve SCW (see Additional file 3: Table S2). The significance of the excretion of typically endogenous proteins must be left open to future enquiry. Given *S. mansoni* miracidia’s ability to differentiate between highly and less infected snails it may be possible that the release of these proteins attracts miracidia [70]. Future research, such as bioassays with specific proteins, should identify whether these proteins or their comprising peptides act as attractants for miracidia.

Our GO enrichment analysis of susceptible *B. glabrata* ESPs indicated greatest enrichment of proteins involved in hydrolase, catalytic and extracellular activity (see Fig. 4b and Table 2). Susceptible *B. glabrata* release zinc metalloproteinase, which is involved in mitigating tissue damage and inflammation [71]. Its presence may suggest the molluscan host is attempting to minimise *S. mansoni* damage. It is difficult to determine the significance of mucin-like proteins involved. The immunological role of mucins in *B. glabrata* has been subject to little investigation [72, 73]. Some notable digestive hydrolases detected in the susceptible SCW include cysteine peptidases [66, 74]. Notable cysteine peptidases include cathepsin B and cathepsin L, the latter of which has been identified in susceptible *B. glabrata* SCW [75]. While cathepsin B correlates with resistance to infection, previous studies have identified cathepsin L-like gene upregulation in *B. glabrata* susceptible to infection from the intestinal fluke *Echinostoma caproni* [76]. Similar responses to *S. mansoni* infection have not been confirmed. Phospholipase is a defensive enzyme vital to superoxide production and oxidase activation [76].

The presence of phospholipase is not necessarily inconsistent with susceptibility. An analysis from another species of *Biomphalaria*, *Biomphalaria pfeifferi*, has suggested that phospholipase activity is most prevalent after three days of infection [77]. This indicates correlation with relatively prolonged presence of *S. mansoni*. Glutathione S-transferase is an antioxidant and given its presence in *S. japonicum* endogenous ESPs it appears to be relatively ubiquitous [26, 55]. Analyses of *E. caproni*-infected *B. glabrata* have identified an almost three-fold increase in mRNA for detoxifying enzymes such as glutathione S-transferase two days post-infection. It is estimated at this stage the parasite has been encapsulated and the enzyme is trying to diminish oxidative stress [78]. Little has been published about the other proteins identified in susceptible *B. glabrata*. Fewer proteins identified as immunologically significant were identified within the susceptible snails, consistent with expectations.

The analysis of susceptible *B. glabrata* ESPs using a reference *S. mansoni* proteome database revealed several proteins, such as G3PDH (see Table 3), which is prominent in host-parasite interactions [26, 79]. Combined with cysteine peptidases, G3PDH is essential in the protection of *S. mansoni* due to its involvement in gene expression [80]. It is a key component of the glycolytic pathway and has been focussed on as a vaccine candidate [81, 82]. *Schistosoma mansoni* G3PDH was supported by three peptides in this study, one of which shares the same sequence (i.e. K.LTGMAFR.V) with *B. glabrata* G3PDH. While the presence of *S. mansoni* G3PDH was confirmed, the presence of *B. glabrata* G3PDH can only be speculated. Egg protein CP391B has been identified exclusively in the sporocyst stage of *S. japonicum* and indicates successful infection [83]. The potential immunosuppressive properties of this protein have not been studied yet. Phosphoribosyltransferase has been identified as essential to nucleotide metabolism, though nicotinate phosphoribosyltransferase has yet to be subject to much analysis [84, 85]. The specific role of proline-serine-threonine phosphatase interacting protein in *S. mansoni* is still unknown. The seven other identified proteins were all novel.

Several PKCs from *B. glabrata* were identified to be interacting with neuropathy targeted esterase and Smp_142140.1 from *S. mansoni*. PKCs are involved in such processes as reparation of damaged tissue and parasite termination through the regulation of hydrogen peroxide [28, 86]. PKC receptors are upregulated most rapidly in resistant snails within 5–10 hours of exposure to miracidia as opposed to susceptible snails, which can take several days to respond [28], suggesting the need for upregulation of PKCs in the development of resistance. These two *S. mansoni* proteins could be targets of *B. glabrata* PKC. Serine threonine kinases have been identified in the RNA of haemocytes in *B. glabrata*; however, besides that little is known about its function in the interaction with *S. mansoni* [87]. Both PKC and serine threonine protein kinase are activated by diacylglycerol [88], which indicates the potential association of these two proteins in response to infection. There are gaps in our understanding of the immunological significance or functions of the other kinases in *B. glabrata* observable in Fig. 5a.

Several proteins present in the PPI conducted with reference to the *S. mansoni* proteome (see Fig. 5b) have been identified in previous studies where proteins isolated from miracidia developing into sporocysts were fractioned by SDS-PAGE. These include fructose-bisphosphate aldolase, transketolase, triosephosphate isomerase, enolase and glucose-6-phosphate isomerase [22, 89]. Furthermore, nicotinamide has also been identified in the sporocysts of *S. mansoni* [90]. Some of these proteins, such as fructose-biphosphate aldolase, triosephosphate dehydrogenase and glucose-6-phosphate dehydrogenase have been identified as glycolytic enzymes, similar to G3PDH, which is also involved in the transition of miracidia to sporocysts [22, 91, 92]. This indicates that these proteins, or other uncharacterised proteins, may function as markers of a successful infection for other miracidia to detect, possibly decreasing their likelihood of further infection.

## Conclusions

In this study, fractionated SCW ESPs from susceptible, F1 resistant and naïve *B. glabrata* were analysed using LC-MS/MS to identify proteins significant to *B. glabrata* and *S. mansoni* interactions. The significant modifications of miracidia behaviour were only observed following the addition of naïve and susceptible (although less prominent) *B. glabrata* SCW. F1 resistant *B. glabrata* SCW displayed ESPs corresponding with immunological activity while susceptible *B. glabrata* SCW contained fewer defensive-type enzymes, potentially conferring a weaker resistance to parasite infection. While several ESPs identified with reference to the *S. mansoni* database have well-documented functions in snails or other species, many remain uncharacterised. Our PPI analysis indicated potential proteins relevant to the response of susceptible snails to miracidia and proteins corresponding to sporocyst development. This suggests that they may be acting as deterrents to miracidia. This study identified several protein candidates to further investigate to reveal the interactions between *B. glabrata* and *S. mansoni*. This may facilitate future innovations into preventing the infection of *B. glabrata* snails and inform research into other molluscan hosts.

## Supplementary information

**Additional file 1: Database S1.** The *B. glabrata* protein database used in the proteomic data analysis.

**Additional file 2: Table S1.** Statistical analysis of behavioural bioassays. Two-way ANONA method was used to evaluate the significance of the behavioural modifications.

**Additional file 3: Table S2.** A list of the total proteins, unique proteins and corresponding peptides identified in naïve *B. glabrata* with reference to the *B. glabrata* proteome.

**Additional file 4: Table S3**. A list of the total proteins, unique proteins and corresponding peptides identified resistant *B. glabrata* with reference to the *B. glabrata* proteome.

**Additional file 5: Table S4.** A list of the total proteins, unique proteins and corresponding peptides identified in susceptible *B. glabrata* with reference to the *B. glabrata* proteome.

**Additional file 6: Figure S1.** The representative MS/MS spectrum of *LTGMAFR*, supporting both *B. glabrata* and *S. mansoni* glyceraldehyde-3-phosphate dehydrogenase (G3PDH).

**Additional file 7: Figure S2.** The representative MS/MS of supporting peptides of *S. mansoni* protein identification.

**Additional file 8: Table S5.** A list of the total proteins, unique proteins and corresponding peptides identified in susceptible *B. glabrata* SCW with reference to the *S. mansoni* proteome, and enriched GO terms related to the proteins.

**Additional file 9: Table S6.** The results of PPIs shown in Fig. 5 and the co-expression data export from STRING to support the PPI of *S. mansoni* proteins.

**Additional file 10: Figure S3.** The co-expression levels of *S. mansoni* proteins identified.

## Data Availability

The mass spectrometry proteomics data have been deposited to the ProteomeXchange Consortium via the PRIDE [41] partner repository with the dataset identifier PXD015129. Other datasets analysed in this study are included in this published article and its additional files.

## Abbreviations

ESP: excretory–secretory protein
FDR: false discovery rate
G3PDH: glyceraldehyde-3-phosphate dehydrogenase
GO: gene ontology
LC-MS: liquid chromatography tandem mass spectroscopy
MS/MS: mass spectrometry tandem mass spectrometry
NCBI: National Center for Biotechnology Information
PBS: phosphate-buffered saline
PKC: protein kinase C
PPI: protein-protein interaction
RNA: ribonucleic acid
SCW: snail-conditioned water
SOD: superoxide dismutase
uHPLC: ultra-high performance liquid chromatography
