# Correction to: Comparative study of excretory–secretory proteins released by *Schistosoma mansoni*-resistant, susceptible and naïve *Biomphalaria glabrata*

**DOI:** 10.1186/s13071-022-05439-9

**Published:** 2022-11-11

**Authors:** Conor E. Fogarty, Min Zhao, Donald P. McManus, Mary G. Duke, Scott F. Cummins, Tianfang Wang

**Affiliations:** 1 Genecology Research Centre, University of the Sunshine Coast, Maroochydore DC, QLD 4558 Australia; 2 QIMR Berghofer Medical Research Institute, Brisbane, QLD 4006 Australia

## Correction to: Parasites Vectors (2019) 12:452 10.1186/s13071-019-3708-0

The authors have provided some corrections following the publication of their article [1]; please see their correction below:

We have identified several errors in our analysis of *S. mansoni* miracidia behaviour data. Tortuosity was calculated from the incorrect metric and some velocity data was misplaced during analysis and figure preparation. Correcting these errors does not noticeably affect the changes in miracidia per min or duration. However, the corrected data now indicates that both resistant and infected SCW induce significant decreases in velocity and increases in tortuosity, while formerly neither SCW treatment significantly affected these metrics. In the methods, we’ve also corrected the detail about how long the *B. glabrata* were infected prior to susceptible SCW collection to say three weeks post-infection instead of two weeks post-infection. Furthermore, the framerate was 15 frames per second, rather than 25. Only some sentences in the short sections relating to behaviour should be altered; the rest of the paper, focussed overwhelmingly on proteomic comparison, does not require change. We have provided minor corrections to sentences in Methods Paragraph 3, Results paragraph 1 and Discussion Paragraph 2. Additionally, we have provided a corrected Fig. 2 and Additional file 2: Table S1 to accurately reflect the corrected data.

**Fig. 2:**
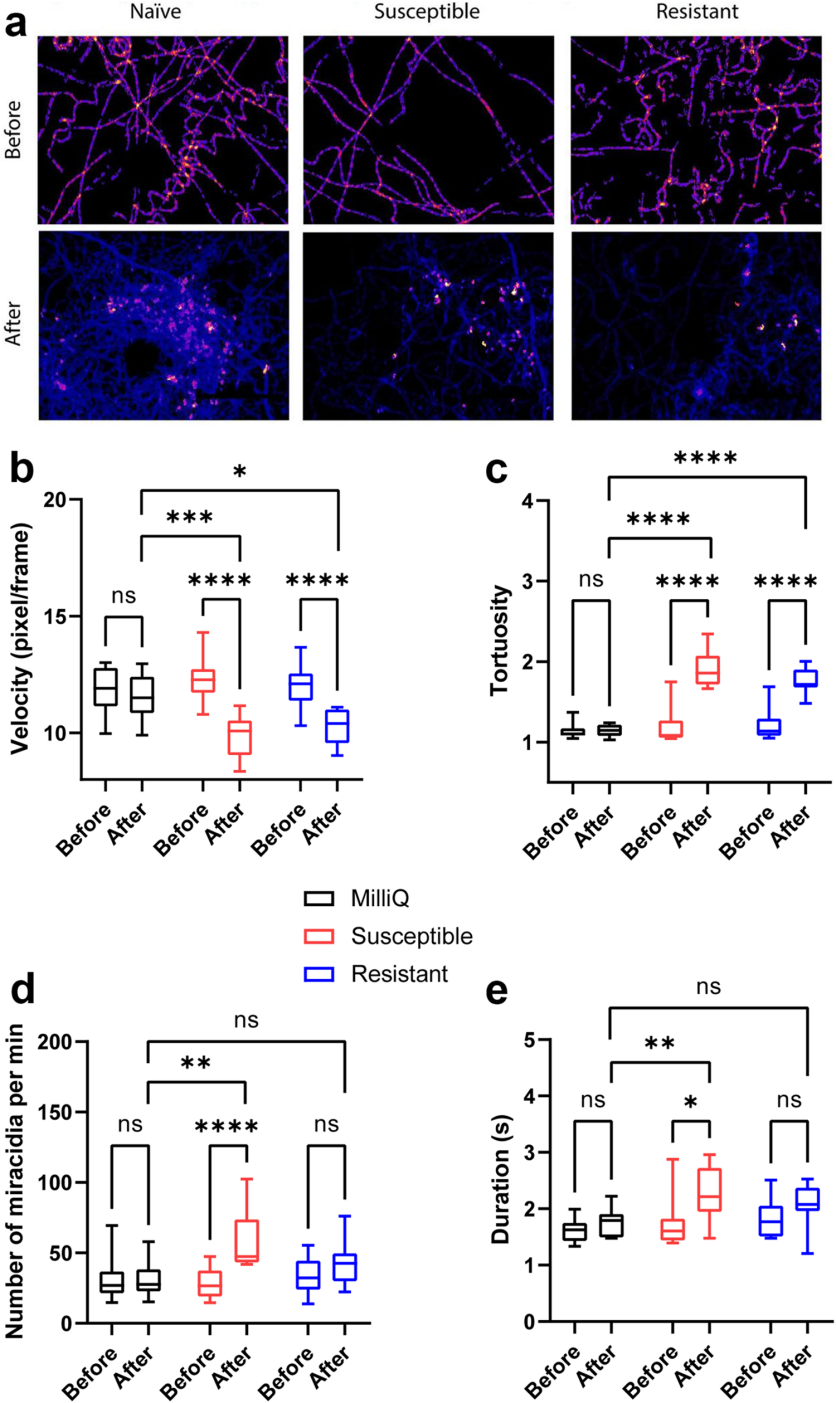
Behavioural modifications of *S. mansoni* miracidia before and after exposure to pH-neutral water, susceptible and resistant SCW. The heatmaps (**a**), linear velocity (**b**), tortuosity (**c**), number of miracidia (**d**) recorded in the FOV within 1 min pre- and post- the addition and duration of miracidia staying in the FOV within 1 min pre- and post- the addition (**e**). A two-way ANOVA test was used to calculate *P*-values: *P < 0.05, **P < 0.01, ***P < 0.001, ****P < 0.0001

Methods Paragraph 3 (Final two sentences):

Briefly, miracidia water aliquots in 200 µl volumes were placed on a petri dish and monitored using an Olympus-CKX41 microscope (Olympus) equipped with an Olympus DPI Digital Microscope Camera DP22 (15 frames per second at 2.8-megapixel image quality). Miracidia behaviour was recorded and monitored for one minute, followed by one minute after the addition of 2 µl of SCW. This process was conducted nine times using naïve, susceptible (from *B. glabrata* exposed to miracidia 3 weeks prior) and F1 resistant *B. glabrata* SCW and one negative control (pH-neutral water used for incubating miracidia).

Results, Paragraph 1:

*Schistosoma mansoni* miracidia behavioural assays

We have previously shown that SCW of naïve *B. glabrata* stimulates significant behavioural changes in *S. mansoni* miracidia, including decreased swimming speed (velocity) and elevated tortuosity, quantity and duration of miracidia presence in the FOV [31]. In this study, we further quantified the changes in *S. mansoni* miracidia behaviour in response to pH-neutral water, susceptible and resistant *B. glabrata* SCW using behavioural bioassays. Figure 2 provides comparative data for the behavioural modifications monitored in the bioassay, with the statistical analysis results shown in Additional file 2: Table S1. Figure 2a displays there are more abundant red and yellow regions in pre-addition heatmaps, indicating relatively slower moving miracidia. The post-addition heatmap of naïve SCW depicts fewer linear motions and a higher proportion of soft blue lines which suggest more tracks in the FOV and quicker circular movements. The post-addition heatmaps of susceptible and F1 resistant SCW only show quicker circular movements, but the changes in the amount of blue lines are less noticeable. The velocity of movement (swimming) of miracidia in three treatments was assessed (Fig. 2b), where significant decreases in velocity were observed following the addition of resistant and susceptible SCW compared to both the control (i.e. pH-neutral water versus susceptible versus resistant) and pre-addition (i.e. pre- versus post- addition within 1 min time frame) (Additional file 2: Table S1). In terms of tortuosity, both resistant and susceptible SCW induced significant increases both within one treatment and between treatments (Fig. 2c). The number of miracidia entering the FOV significantly increased within 1 min post-addition of susceptible SCW, but not after addition of pH-neutral water or resistant SCW (Fig. 2d). The duration of miracidia staying in the FOV was also significantly elevated exclusively after the addition of susceptible SCW (Fig. 2e). In summation, susceptible SCW induced comparable changes to naïve SCW in all behaviour metrics, including decreased velocity and increased tortuosity, miracidia quantity and duration of presence, while resistant SCW only induced decreased velocity and increased tortuosity.

Discussion paragraph 2:

In the presence of SCW, miracidia tend to increase their angular velocity while slightly decreasing linear velocity [31, 53]. We had previously shown that naïve *B. glabrata* SCW significantly reduced miracidia velocity and elevated tortuosity by approximately 20% and 70%, respectively [31]. As shown in Fig. 2b, c, susceptible and F1 resistant *B. glabrata* SCW both induced comparable significant changes in miracidia linear velocity and tortuosity. The response of miracidia to pH-neutral water is consistent with expectations, as this water had not been exposed to any *B. glabrata*. The quantity of miracidia present following addition of susceptible SCW increased significantly when compared to pre-SCW or post-pH-neutral water addition, indicating a possible attraction effect (Fig. 2d). However, the increase in activity was remarkably weaker than that of naïve SCW, which produced an increasing magnitude of about 4-fold [31]. This suggests that susceptible *B. glabrata* at three-weeks post-infection might still release attractant(s) yet at a lower concentration than naïve snails. A similar change was observed for the duration of miracidia staying in the FOV. In contrast, there was no significant change in miracidia quantity or duration of presence post-resistant SCW addition, possibly due to decreased attractant concentration compared to susceptible *B. glabrata*, or counteraction from potential repellents. Therefore, it appears that susceptible SCW causes decreased velocity and increased tortuosity and the duration and quantity of miracidia presence, while resistant SCW only affects tortuosity and velocity. This requires more experimental verification in future studies.

## Supplementary Information

**Additional file 2: Table S1.** Statistical analysis of behavioural bioassays. Two-way ANONA method was used to evaluate the significance of the behavioural modifications.
