# Supplementary figures and images for: Comparative study of excretory–secretory proteins released by Schistosoma mansoni-resistant, susceptible and naïve Biomphalaria glabrata
Source: Parasit Vectors. 2019 Sep 14;12:452. doi: 10.1186/s13071-019-3708-0 (PMC6744689; doi:10.1186/s13071-019-3708-0)

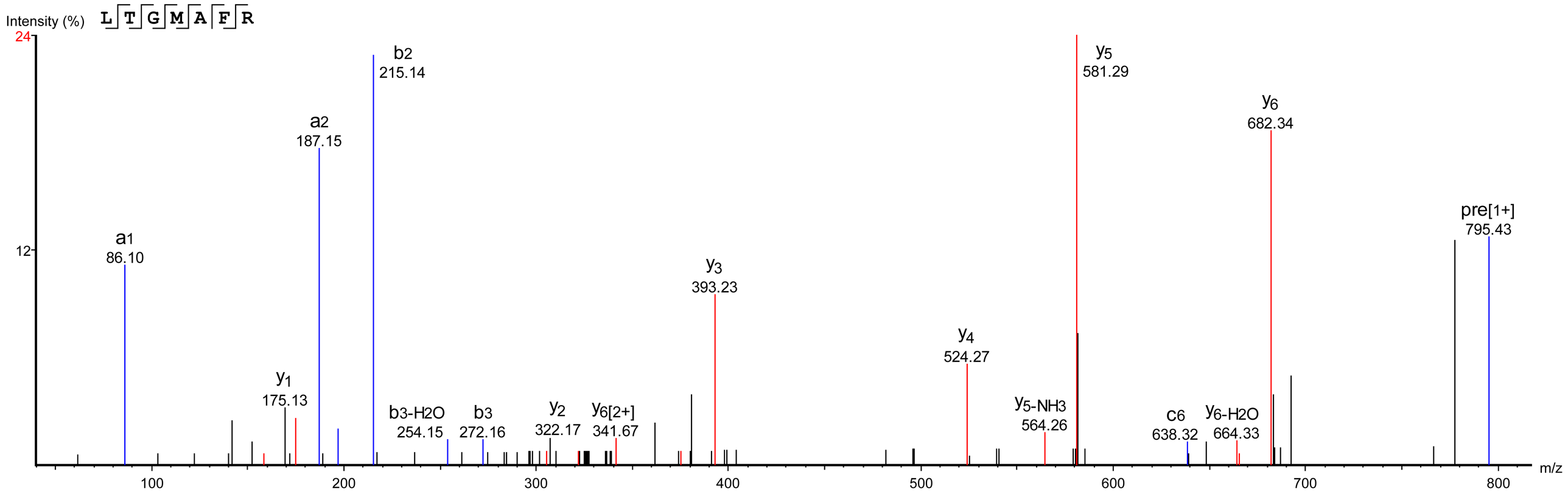

Supplement: Supplementary file 6 — Additional file 6: Figure S1. The representative MS/MS spectrum of LTGMAFR, supporting both B. glabrata and S. mansoni glyceraldehyde-3-phosphate dehydrogenase (G3PDH). [file 13071_2019_3708_MOESM6_ESM.tif]

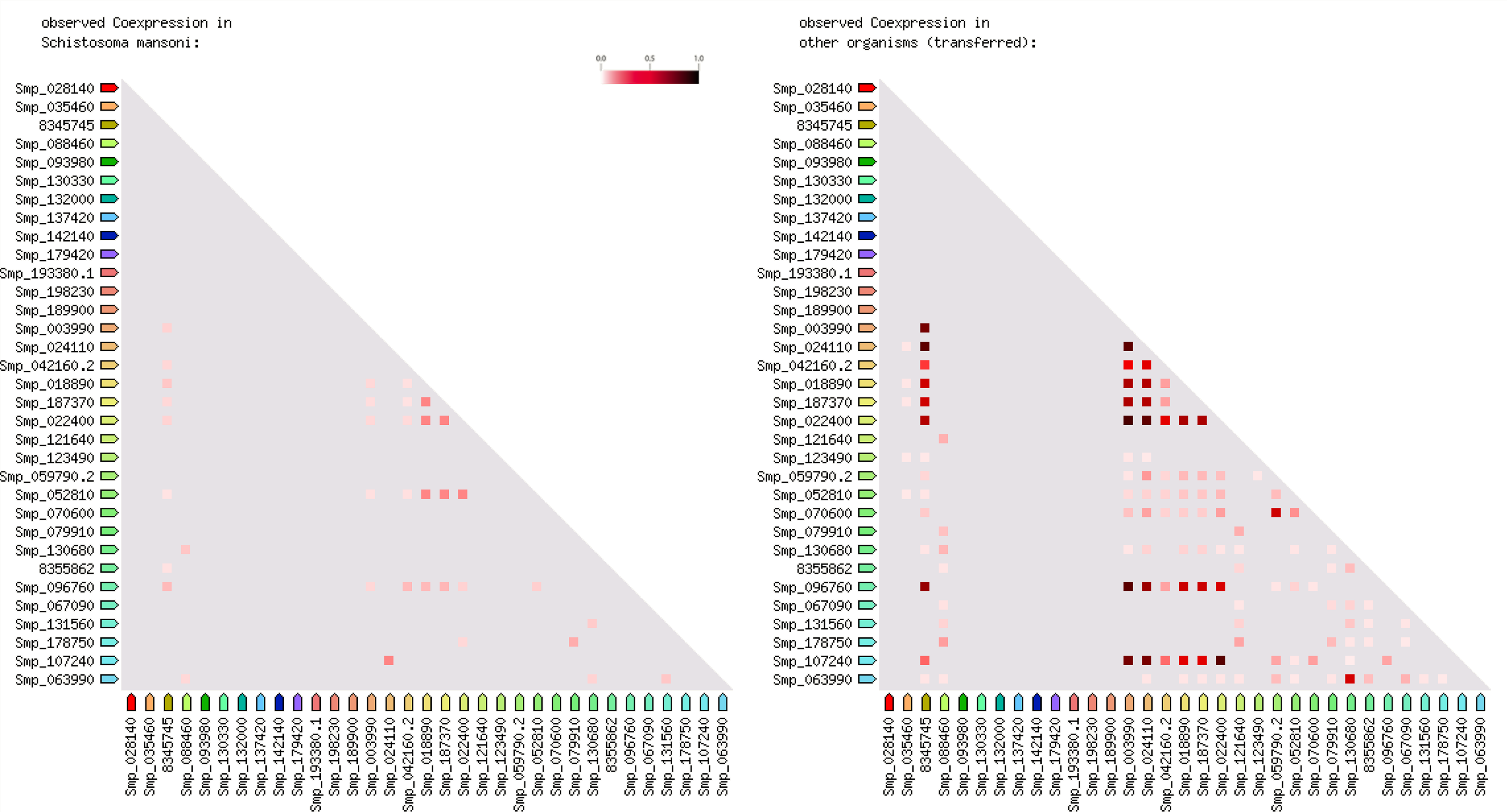

Supplement: Supplementary file 10 — Additional file 10: Figure S3. The co-expression levels of S. mansoni proteins identified. [file 13071_2019_3708_MOESM10_ESM.tif]
