# Supplementary material for: Comparative study of excretory–secretory proteins released by Schistosoma mansoni-resistant, susceptible and naïve Biomphalaria glabrata
Source: Parasit Vectors. 2019 Sep 14;12:452. doi: 10.1186/s13071-019-3708-0 (PMC6744689; doi:10.1186/s13071-019-3708-0)

Smp_035460.1

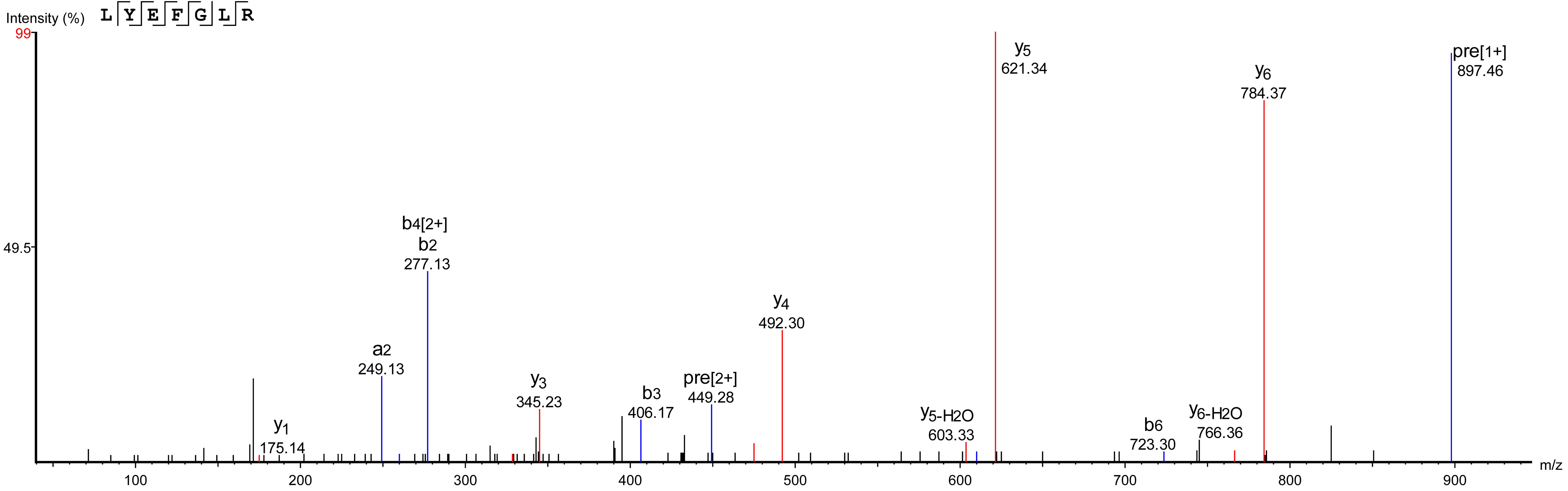
Intensity (%)
L Y E F G L R
99
49.5
y5
621.34
pre[1+]
897.46
y6
784.37
b4[2+]
b2
277.13
y4
492.30
a2
249.13
y3
345.23
pre[2+]
449.28
b3
406.17
y1
175.14
y5-H2O
603.33
b6
723.30
y6-H2O
766.36
100
200
300
400
500
600
700
800
900
m/z

Smp_056970.4

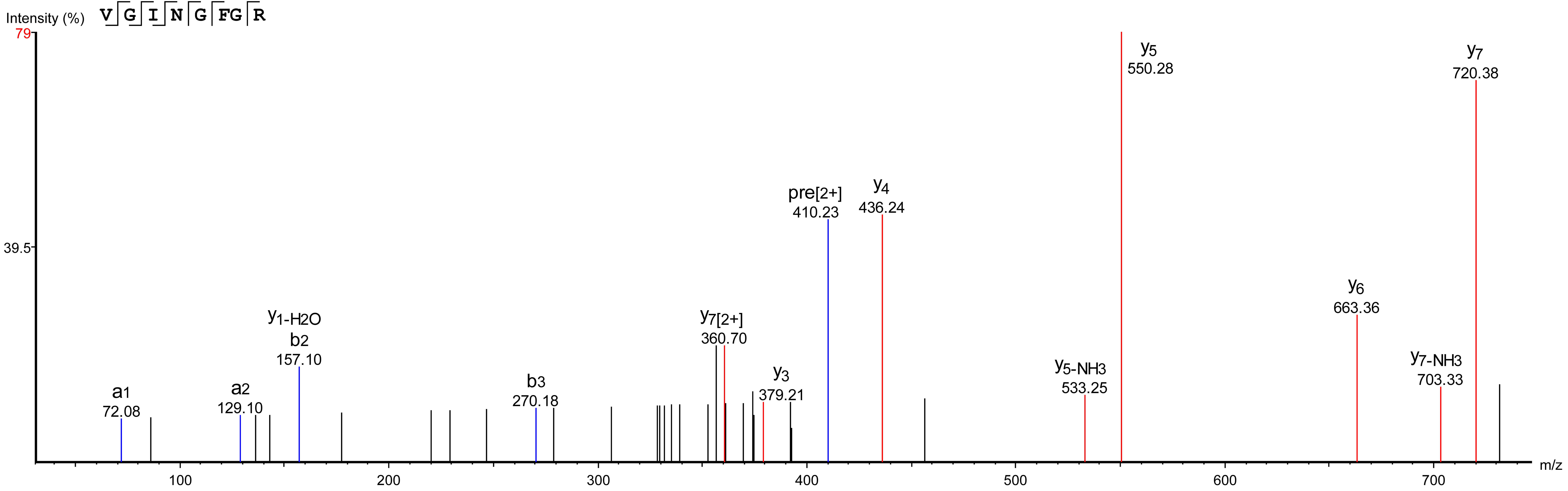
Intensity (%)
V G I N G F G R
79
39.5
y5
550.28
y7
720.38
pre[2+]
410.23
y4
436.24
y6
663.36
y1-H2O
b2
157.10
y7[2+]
360.70
y3
379.21
y5-NH3
533.25
y7-NH3
703.33
a1
72.08
a2
129.10
b3
270.18
100
200
300
400
500
600
700
m/z

Smp_088460.1

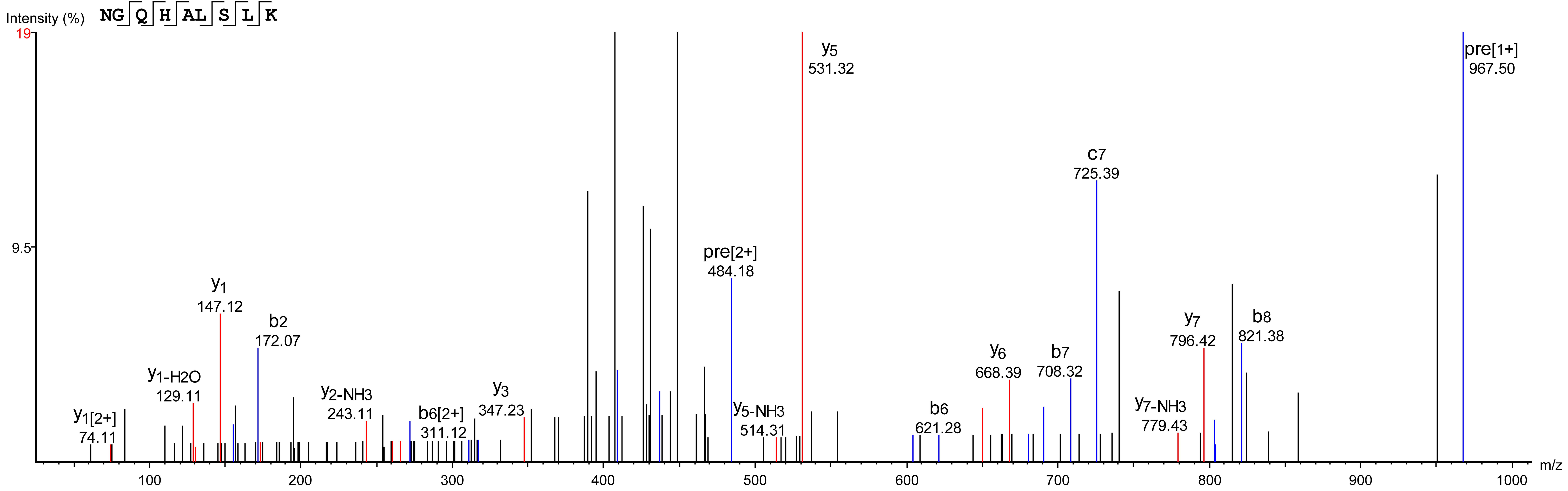

Intensity (%)
NG Q H AL S L K
19
9.5
y1[2+]
74.11
y1-H2O
129.11
y1
147.12
b2
172.07
y2-NH3
243.11
b6[2+]
311.12
y3
347.23
pre[2+]
484.18
y5-NH3
514.31
y5
531.32
b6
621.28
y6
668.39
b7
708.32
c7
725.39
y7-NH3
779.43
y7
796.42
b8
821.38
pre[1+]
967.50
100
200
300
400
500
600
700
800
900
1000
m/z

Smp_093980.1

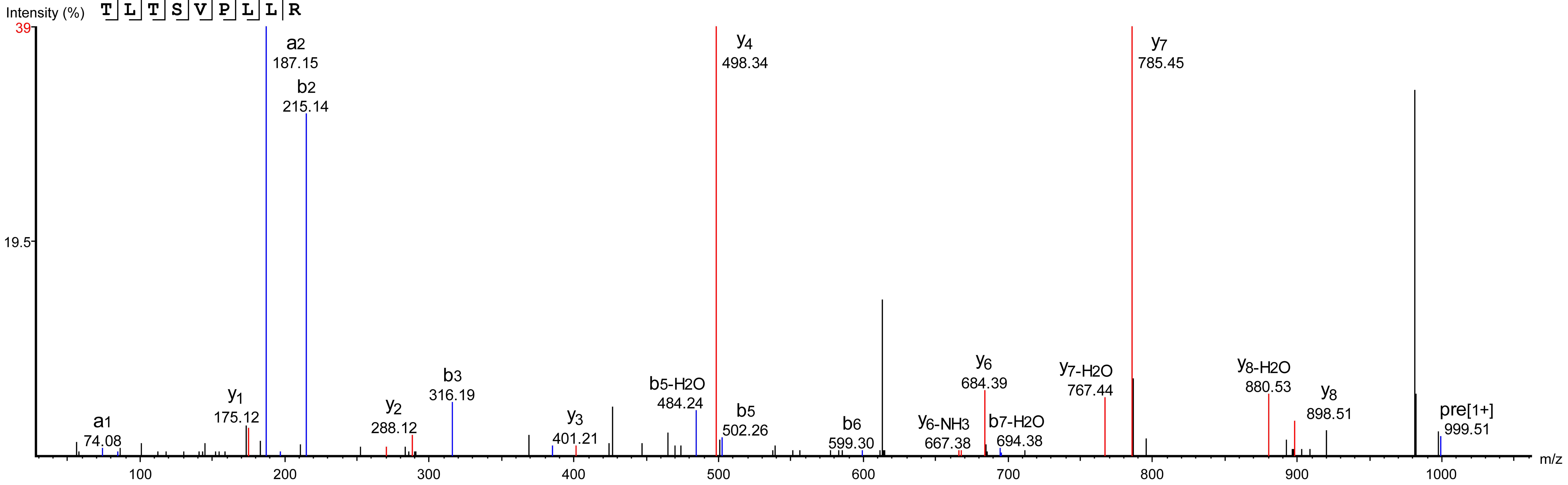

Intensity (%)
T L T S V P L L R
39
19.5
a1
74.08
y1
175.12
a2
187.15
b2
215.14
y2
288.12
b3
316.19
y3
401.21
b5-H2O
484.24
y4
498.34
b5
502.26
b6
599.30
y6-NH3
667.38
y6
684.39
b7-H2O
694.38
y7-H2O
767.44
y7
785.45
y8-H2O
880.53
y8
898.51
pre[1+]
999.51
100
200
300
400
500
600
700
800
900
1000
m/z

Smp_130330.1

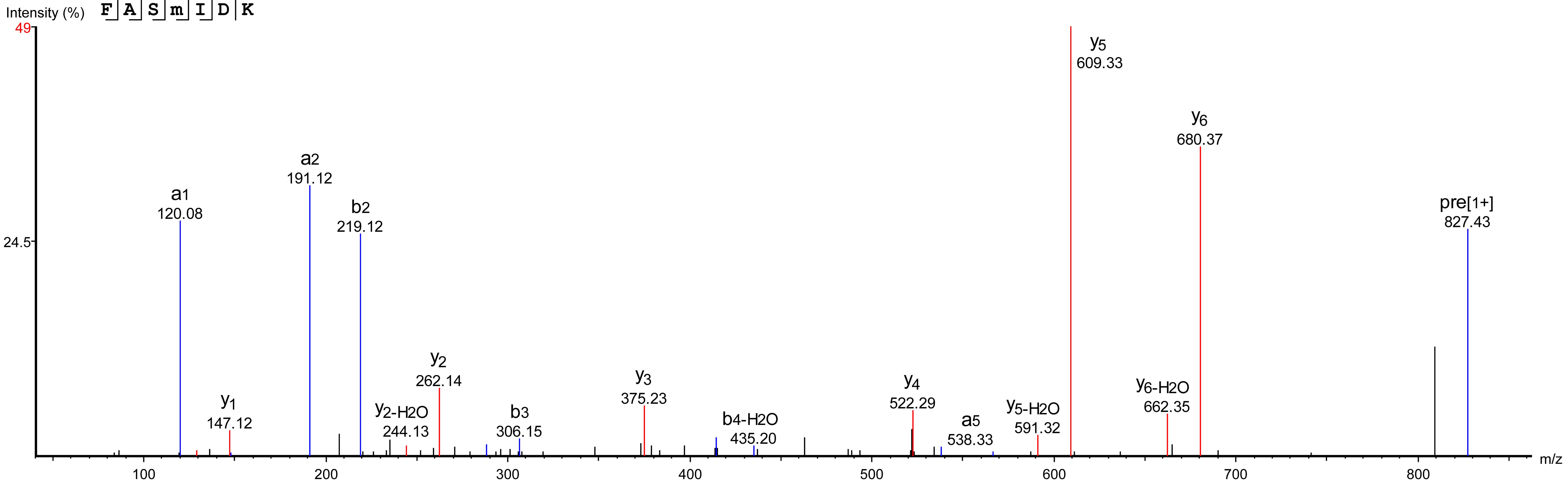
Intensity (%)
F A S m I D K
49
24.5
a1 120.08
y1 147.12
a2 191.12
b2 219.12
y2-H2O 244.13
y2 262.14
b3 306.15
y3 375.23
b4-H2O 435.20
y4 522.29
a5 538.33
y5-H2O 591.32
y5 609.33
y6-H2O 662.35
y6 680.37
pre[1+] 827.43
100
200
300
400
500
600
700
800
m/z

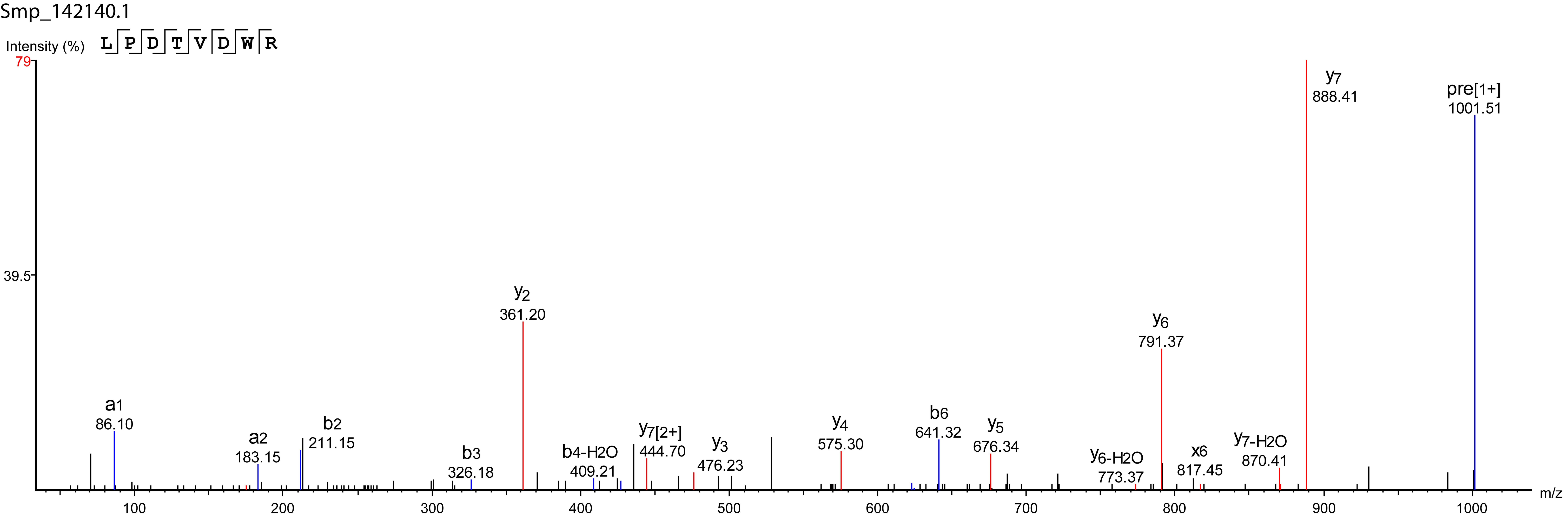
Smp_142140.1
Intensity (%)
L P D T V D W R
79
39.5
y7 888.41
pre[1+] 1001.51
y2 361.20
y6 791.37
a1 86.10
a2 183.15
b2 211.15
b3 326.18
b4-H2O 409.21
y7[2+] 444.70
y3 476.23
y4 575.30
b6 641.32
y5 676.34
y6-H2O 773.37
x6 817.45
y7-H2O 870.41
100
200
300
400
500
600
700
800
900
1000
m/z

Smp_179420.1_1

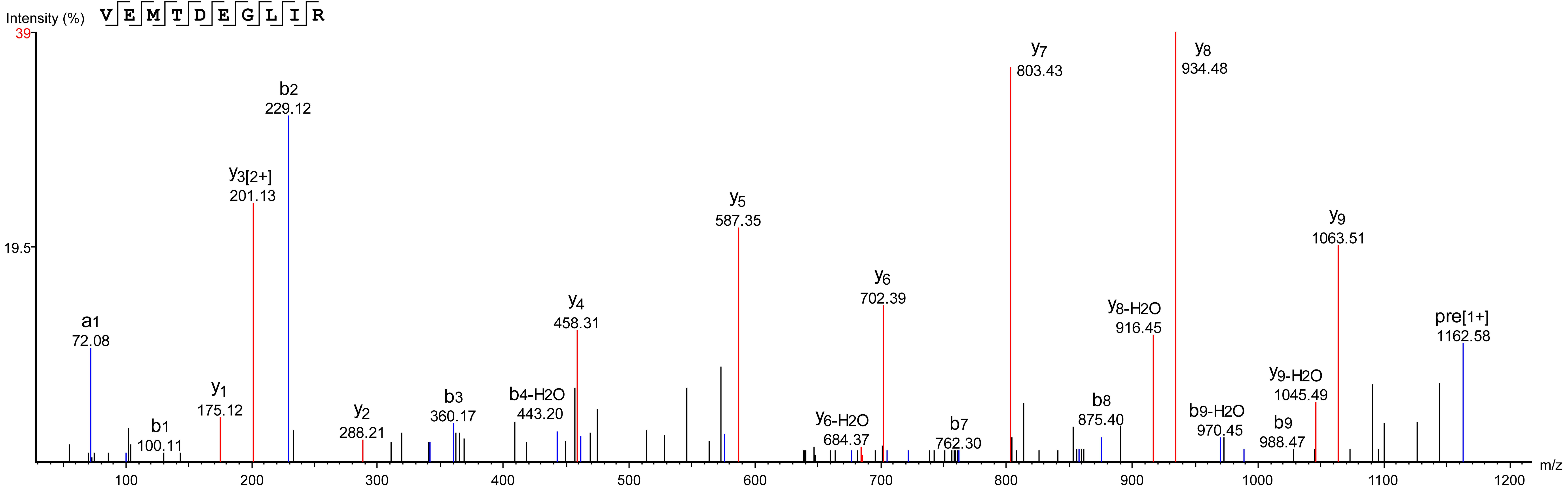

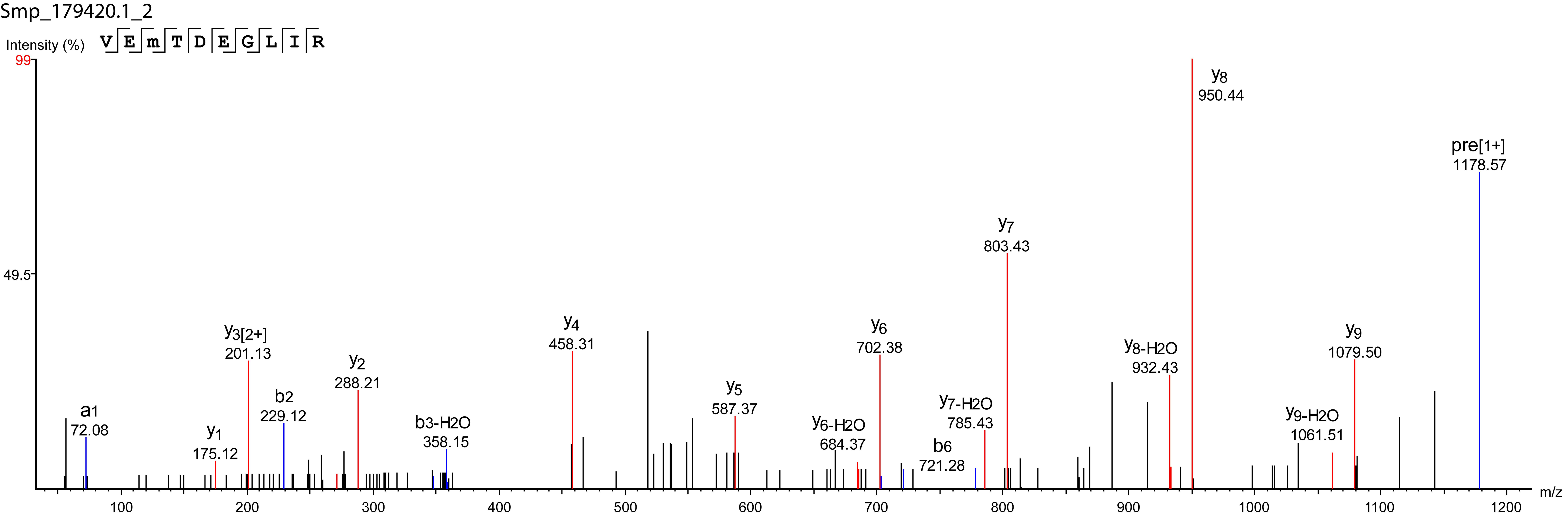

Smp_179420.1_2
Intensity (%)
V E m T D E G L I R
99
49.5
a1 72.08
y1 175.12
y3[2+] 201.13
b2 229.12
y2 288.21
b3-H2O 358.15
y4 458.31
y5 587.37
y6-H2O 684.37
y6 702.38
b6 721.28
y7-H2O 785.43
y7 803.43
y8-H2O 932.43
y8 950.44
y9-H2O 1061.51
y9 1079.50
pre[1+] 1178.57
100
200
300
400
500
600
700
800
900
1000
1100
1200
m/z

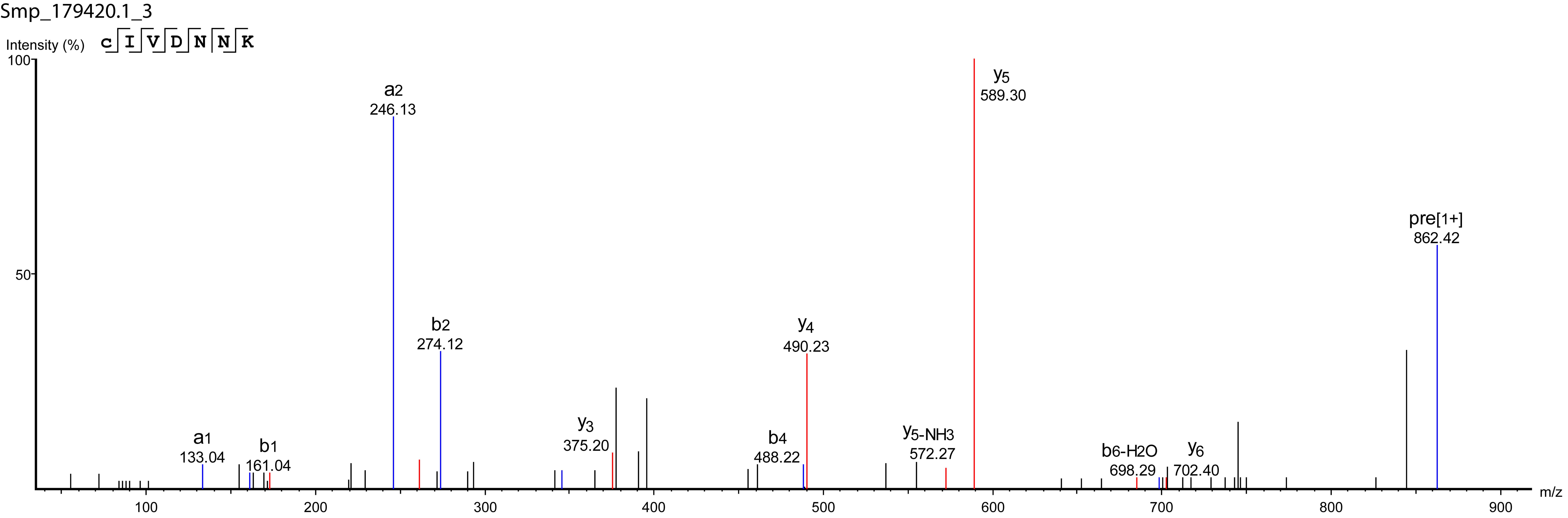
Smp_179420.1_3
Intensity (%)
C I V D N N K
100
50
a1
133.04
b1
161.04
a2
246.13
b2
274.12
y3
375.20
b4
488.22
y4
490.23
y5-NH3
572.27
y5
589.30
b6-H2O
698.29
y6
702.40
pre[1+]
862.42
100
200
300
400
500
600
700
800
900
m/z

Smp_193380.1

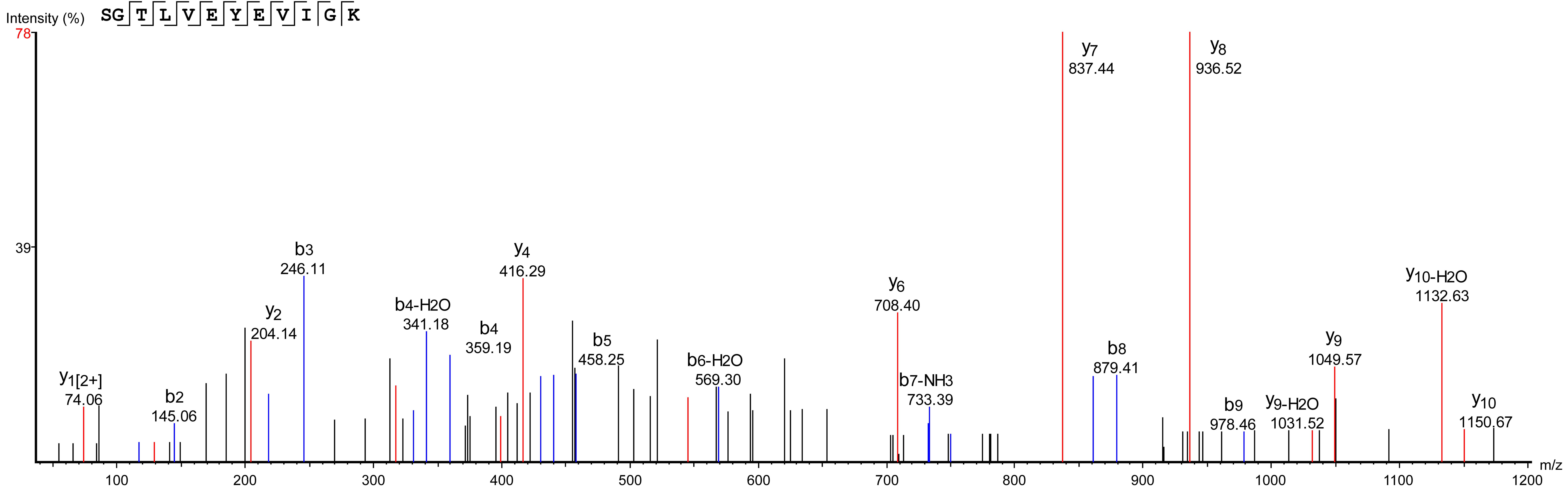

Intensity (%)
SG T L V E Y E V I G K
78
39
y7 837.44
y8 936.52
b3 246.11
y4 416.29
y6 708.40
y10-H2O 1132.63
y2 204.14
b4-H2O 341.18
b4 359.19
b5 458.25
b6-H2O 569.30
b8 879.41
y9 1049.57
y1[2+] 74.06
b2 145.06
b7-NH3 733.39
b9 978.46
y9-H2O 1031.52
y10 1150.67
100
200
300
400
500
600
700
800
900
1000
1100
1200
m/z

Smp_202190.1_1

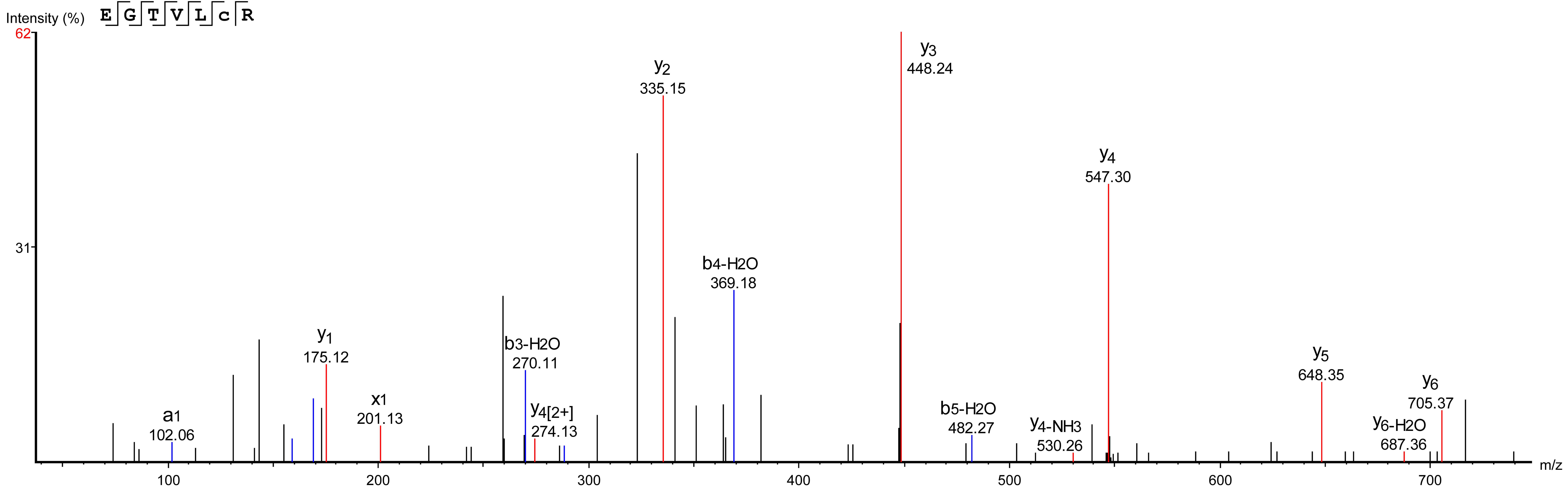

Smp_202190.1_2

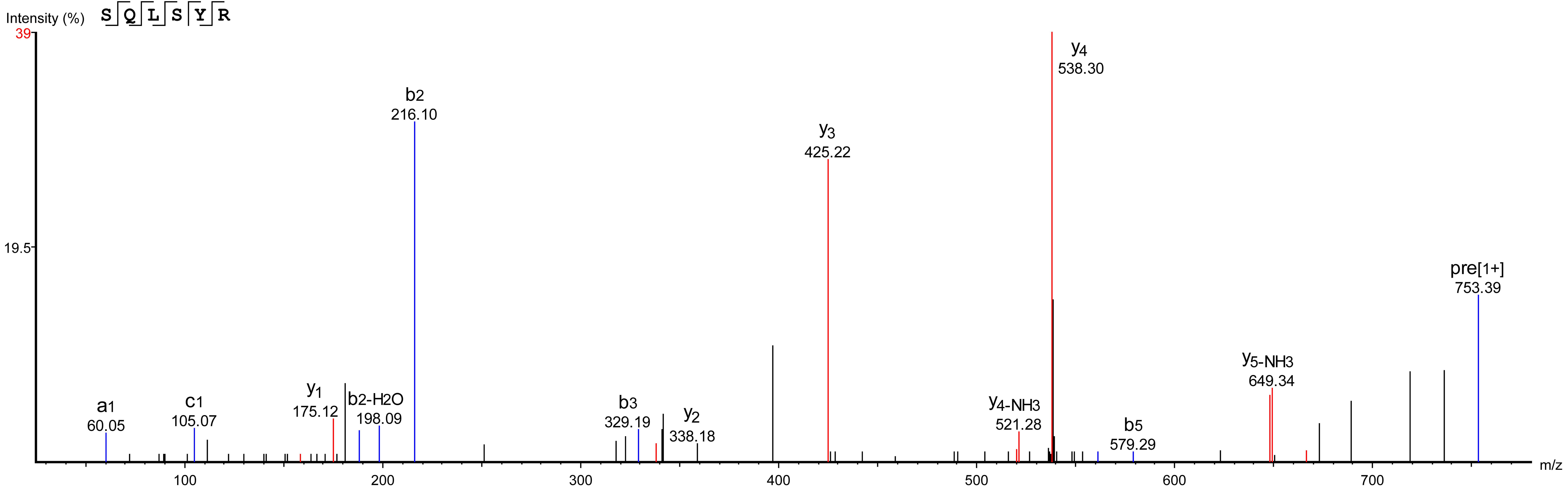

Supplement: Supplementary file 7 — Additional file 7: Figure S2. The representative MS/MS of supporting peptides of S. mansoni protein identification. [file 13071_2019_3708_MOESM7_ESM.pdf]
